# Aligning social networks and co-designed visions to foster systemic innovation in the Alps

**DOI:** 10.1007/s10113-023-02099-y

**Published:** 2023-07-28

**Authors:** Victor Blanco, Tobias Luthe, Enora Bruley, Adrienne Grêt-Regamey

**Affiliations:** 1 Planning of Landscape and Urban Systems, ETH Zürich, Zurich, Switzerland; 2 Institute of Science, Technology and Policy, ETH Zürich, Zurich, Switzerland; 3 The Oslo School of Architecture and Design, Oslo, Norway; 4 Laboratoire d’Ecologie Alpine, Université Grenoble Alpes, Grenoble, France

**Keywords:** Governance innovation, Regional innovation system, Social network analysis, Social-ecological-technical system, Resilience

## Abstract

Mountain regions face substantial challenges and opportunities arising from global change. The capacity of mountain regions for (systemic) innovation will be determinant to the success of system transformations envisioned by social actors of mountain communities. By analysing the social networks of two regions in the Alps and relating them to desired future visions of sustainable regional development, we provide insights about innovative capacities in mountain regions and propose how to strengthen these capacities in order to support regional transformations. In particular, we analyse innovation systems’ collaboration networks to evaluate the innovative capacity of communities, co-construct visions with mountain stakeholders, and assess innovation requirements to meet these visions. Accordingly, we propose changes to the collaboration networks for addressing regional innovation system (RIS) capacities to help achieve visions through systemic innovation. Our analysis furthers the understanding on the type and magnitude of regional innovation system changes required to more effectively address desired transformative visions in mountain regions. We close by discussing implications of our approach for the assessment of innovative capacity and the transformation of mountain regions.

**Graphical Abstract:**

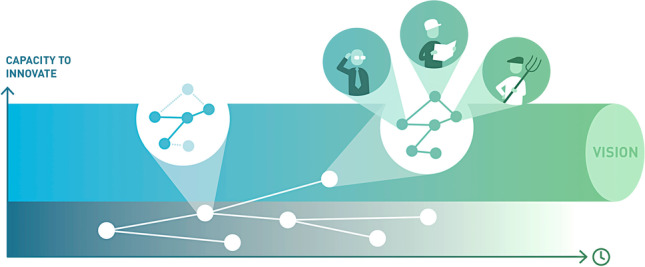

**Supplementary Information:**

The online version contains supplementary material available at 10.1007/s10113-023-02099-y.

## Introduction

Mountains are globally among the most sensitive regions to climatic and socio-economic change, and experience impacts earlier and in a more pronounced manner than most other regions (Kohler et al. 2014; Wehrli 2014). An expected high impact of climate change on natural hazards, water and snow resources, and the impossibility for many plant species to migrate will result in higher vulnerability of transport and energy infrastructure, tourism, agriculture, and biodiversity in mountain regions (Adler et al.^ 2022^). Global change is also increasing the vulnerability of mountain communities to several natural and socio-economic risks (Grover et al. 2015). In contrast, new opportunities for the sustainable development of mountain regions arise from the emergence of alternative economic models (e.g. circular economy, a-growth, degrowth) (Wei et al. 2009; van den Bergh and Kallis 2012; Zhou et al. 2017; Ranta et al. 2018), the creation of new markets (e.g. carbon trading, compensation for ecosystem services), and new consumption patterns that favour high-value goods and services like eco-tourism, freshwater, off-season vegetables, or medicinal plants (Grover et al. 2015). Addressing these threats and opportunities will require transformating these social-ecological-technical systems (McPhearson et al. 2021).

Successfully transforming mountain social-ecological systems is dependent on the capacity of its’ stakeholders to generate systemic and socially desirable innovations (Rodima-Taylor et al. 2012; Aase et al. 2013; Mulgan and Leadbeater 2013; Colvin et al. 2014; Coenen et al. 2015; Cohen et al. 2016; Tödtling and Trippl 2018). This innovative capacity is, together with the capacity of a mountain community or region to respond to disturbances, necessary for building resilience (Luthe and Wyss 2016). Innovative capacity can be defined as the conditions that enable individuals and collectives to create and harness technical and social (including governance) innovation (Cohen et al. 2016). Innovative capacity can be further distinguished by unlocking capacity (phasing-out path dependency), the actual embedding of novelties (transformative capacity), and the coordination of multi-actor processes (orchestrating capacity) (Hölscher et al. 2019). Hence, mountain systems with greater innovative capacity, in which local and regional actors are able to steer this capacity — in so called regional innovation systems (RIS) — will be more likely to achieve successful, envisioned transformations.

How can we gain concrete insights into innovative capacities of mountain RIS — and how would we, based on such insights, strengthen communities in order to support local to regional transformations towards desired visions? This paper contributes with insights on two mountain regions’ capacities to innovate, and proposes how to strengthen such capacities in order to support cross-scalar, local to regional transformations. We relate insights on innovative capacities (gathered through social network analysis (SNA)) to desired stakeholders’ visions to understand how to strengthen the innovative capacity of two mountain regions of the Alps. We start by laying out the conceptual framework that describes the differentiated potential for innovation in mountain regions and present the building blocks to analyse and guide the strengthening of innovative capacities in mountain RIS. We then empirically assess the innovative capacities and requirements by (1) analysing the network structure of the RIS (i.e. actor, interaction, resource, and institutional elements); (2) co-constructing visions with representative stakeholder groups and evaluating the level of innovation required to achieve vision elements; and (3) proposing network adjustments to strengthen their RIS in the direction of achieving their visions through systemic innovation. Finally, we discuss how our empirical analysis can inform the transformation of mountain collaboration networks in ways that support systemic innovation towards desired visions — and its limitations.

## Theory

### Mountain systems as regional innovation systems

Innovations are induced and implemented by networks of interacting organisations and individuals integrated within an institutional context (van de Ven 1986; Swan et al. 1999; van der Valk et al. 2011a, b). Therefore, actors, their interactions, institutions, and infrastructure (including physical, knowledge, and financial resources) have been suggested as the structural elements of the systems within which innovations are generated and implemented (i.e. innovation systems) (see Wieczorek and Hekkert 2012). The concept of innovation system offers a theoretical lens to study the rise of new (socio-technical) systems that provide alternative means to fulfil societal functions (e.g. food production and consumption, energy supply and use, or mobility). Jacobsson and Bergek (2011) propose innovation system analyses as a tool for identifying systemic weaknesses. Systemic weaknesses or failures are the factors obstructing the development and functioning of innovation systems, and subsequently having a negative impact on the direction and speed of innovation (Klein Woolthuis et al. 2005). In other words, systemic weaknesses hamper innovative capacity and, thus, performance.

While national agencies and actors can be very influential to the trajectories of innovations, local and regional development initiatives can support the creation and diffusion of innovation within regions embedded in wider national and global systems. Grumbine and Xu (2021) present several examples of innovative adaptations being explored and pursued in mountain social-ecological systems. The innovative capacity of local and regional actors can be stimulated through interventions to build or strengthen regional innovation systems (RIS) (Asheim and Isaksen 2002; Tödtling and Trippl 2005). In RIS, collective learning, innovation, and entrepreneurial activities are shaped by inter-organisational networked interactions, knowledge, and policy-supported infrastructures and socio-cultural and institutional environments.

The RIS framework proposes that intensifying the public–private cooperation, trust, and knowledge transfer can strengthen the capacity to innovate of individual private and public organisations in the region in the medium to long term (Cooke et al. 1997). RIS policy aims at strengthening such cooperation and knowledge flows between local firms and knowledge organisations, and at supporting their capacity to absorb and exploit knowledge. This can play a crucial role in systemic innovation (Elzen and Wieczorek 2005), resulting in additional new impulses for social-ecological-technical development (Gerstlberger 2004; Sarkis et al. 2010; Lowitt et al. 2015).

While innovation has an important role to play in the transformation of social-ecological-technical systems, it is certainly not a silver bullet. Innovation processes do not always lead to improved system states. Furthermore, they may face challenges along the way, which pertain to power, lack of coordination, and personal or institutional interests and priorities. Tensions around politics and power inequalities may hinder or slow down desired transformation. The RIS framework addresses these challenges in looking at institutional elements of the system — and our chosen methods, including social network analysis and qualitative visioning workshops, are adequate to reveal such potential barriers to successful innovation.

Different typologies of RIS exist that may aid understanding innovative capacities in mountains (see Doloreux 2002). Relevant to innovation governance (i.e. how innovation processes are managed and controlled) are Cooke’s (2004) RIS types. He distinguishes between grassroots, network, and dirigiste RIS. Isaksen and Trippl (2016a) distinguish three types of RIS according to their capacity for endogenous regional transformation. These are ordered by decreasing capacity:

- *Organisationally thick and diversified RIS* feature ‘a relatively large number of different firms, a heterogeneous industrial structure, and a critical mass of knowledge and supporting organisations that facilitate innovation in a wide range of economic and technological fields’. They are often found in core regions like metropolitan areas and advanced technology regions.
- *Organisationally thick and specialised RIS* ‘host strong clusters in one or few industries only. Strong industrial specialisation is further reinforced by support structures that are well adapted to the region’s narrow industrial base’. They are characteristic of industrial districts, old industrial areas, or specialised university campus-towns.
- *Organisationally thin RIS* ‘have only few knowledge and support organisations, and none or only weak developed clusters’. These are typical of peripheral regions.

Economically peripheral regions are characterised by traditional and resource-based industries dominated by small- and medium-sized enterprises, low levels of R&D, and limited or lacking support infrastructure (Tödtling and Trippl 2005; Virkkala 2007; Isaksen 2015). Many such regions are often also geographically peripheral. The relative isolation of mountain areas, their loss of highly skilled and educated people to cities, and a relatively low population size and density hinder the creation of a ‘critical mass’ characteristic of thick and diverse RIS (Fritsch and Slavtchev 2011; de Noni et al. 2018). Therefore, mountain regions are often considered economically and geographically peripheral (Mayer and Baumgartner 2014). Yet, recent historical studies show that mountain regions have been places of innovation, both as adaptation to harsh and changing conditions, and as preparation for future (economic) opportunities, e.g. in tourism (Mathieu and Vester 2009; Denning 2015).

Innovation in mountain regions requires some degree of ‘concentration, path dependency, external inputs, and/or accessibility’ (Eder 2018). Factors such as the connection to extraregional actors, formalised cooperation, the arrival of external actors, the capacity of smaller jurisdictions to adapt faster to the needs of regional economic actors, social learning, and strengthening knowledge attraction and absorption capacities through policy support have shown to foster innovation in the periphery (Isaksen and Trippl 2016b; Matous and Todo 2018; Trippl et al. 2018; Eder 2018; Eder and Trippl 2019; Isaksen 2015). Peripheral spaces can also offer opportunities for experimentation, as they are somewhat shielded from mainstream ideas and accelerating economic pressures (Grabher 2018; Mayer 2020). Shearmur (2017) and Mayer (2020) point out that peripheries are suitable to generate ‘slow’ innovations, even if scaling up is much more restricted to agglomerations.

An example for peripheral mountain innovation networks that are an integral part of RIS is tourism destination management organisations (DMO). Mountain regions where single communities strengthen their collaboration with others on a regional (destination) scale have shown to have higher innovative capacities (Luthe et al. 2012).

Overall, assessing the RIS in which mountain communities are embedded may provide valuable information on their innovative capacities. By assessing their collaborative structure, we gain understanding about structural properties as starting points to propose adjustments for increasing innovative capacities, which then allow for transformative action towards desired visions.

### Guiding systemic innovation towards desired visions

Over the last decade, there has been an increasing recognition that innovation policy needs to focus on innovation as a participatory and inclusive search process at the system level that is guided by social and environmental objectives (Schot and Steinmueller 2018; Tödtling and Trippl 2018). Transformative systemic changes towards sustainability can be described and analysed using an innovation systems framework that engages with issues of directionality (Schlaile et al. 2017). At its core, the RIS framework understands the region as a network of actors that is built up by regional resources (Asheim and Isaksen 2002). The capacity of regional actors to innovate is strongly determined by their collaboration and resource sharing to achieve shared goals, but may as well be influenced by power struggles and institutions. Consequently, organisations often seek access to knowledge networks that complement their core expertise (Grant and Baden-Fuller 2004), and policy makers draw attention to the prominent role of collaboration networks for innovation (van der Valk et al. 2011a, b; de Noni et al. 2018; Lee 2019). Hence, building directionality into innovation performed in RIS is key if the intention is to support desired transformations.

Addressing complex problems, such as climate change, biodiversity loss, or water insecurity, through systemic innovation relies on multi-stakeholder processes of co-creation, experimentation, and social learning (Dentoni et al. 2018; Elia and Margherita 2018; Matous and Todo 2018). Doing so requires organisation and coordination of governance processes, involving various types of actors in participatory and inclusive processes of vision building and learning (Elzen and Wieczorek 2005; Kemp and Never 2017), where the directionality of innovation is important to guide collaboration and development of social networks (Schot and Steinmueller 2018). Strategically incubating and developing social networks between various types of social actors, simultaneously supporting multiple social processes, is required to foster systemic innovation in communities and regions (Levy and Lubell 2018).

Given the diversity of goals pursued for resilient mountain regions (e.g. livelihood sustainability, quality of life, accessibility), aspiring visions developed collectively by mountain stakeholders can be appropriate to guide the systemic innovation process. Understanding the requirements for innovation to meet a vision will thus be key to guide collaboration network development.

A collaboration network can be seen as a mechanism to strengthen innovative capacities in RIS, while a collective vision is the instrument to guide the development of collaborative relations for systemic innovation. Collaboration networks and visions cross-fertilise each other, and thus need to be looked at in an integrated way. However, few studies consider bringing together assessments of collaboration networks with stakeholder-driven future visions. Some studies have assessed the innovative capacity or performance of constellations of actors by analysing collaboration or innovation networks. Some of them also studied innovation in the context of supporting resilience (Moore and Westley 2011; Kelman et al. 2016; Lee 2018), while others looked into innovative capacity for a general goal, such as climate change adaptation (Luthe et al. 2012; Luthe and Wyss 2016). Conceptually and empirically aligning these analyses with the RIS framework can help to better integrate such assessments into regional innovation policy development and assessment mechanisms (Anderson 2008; Cvitanovic et al. 2016; Hering 2016).

**Fig. 1:**
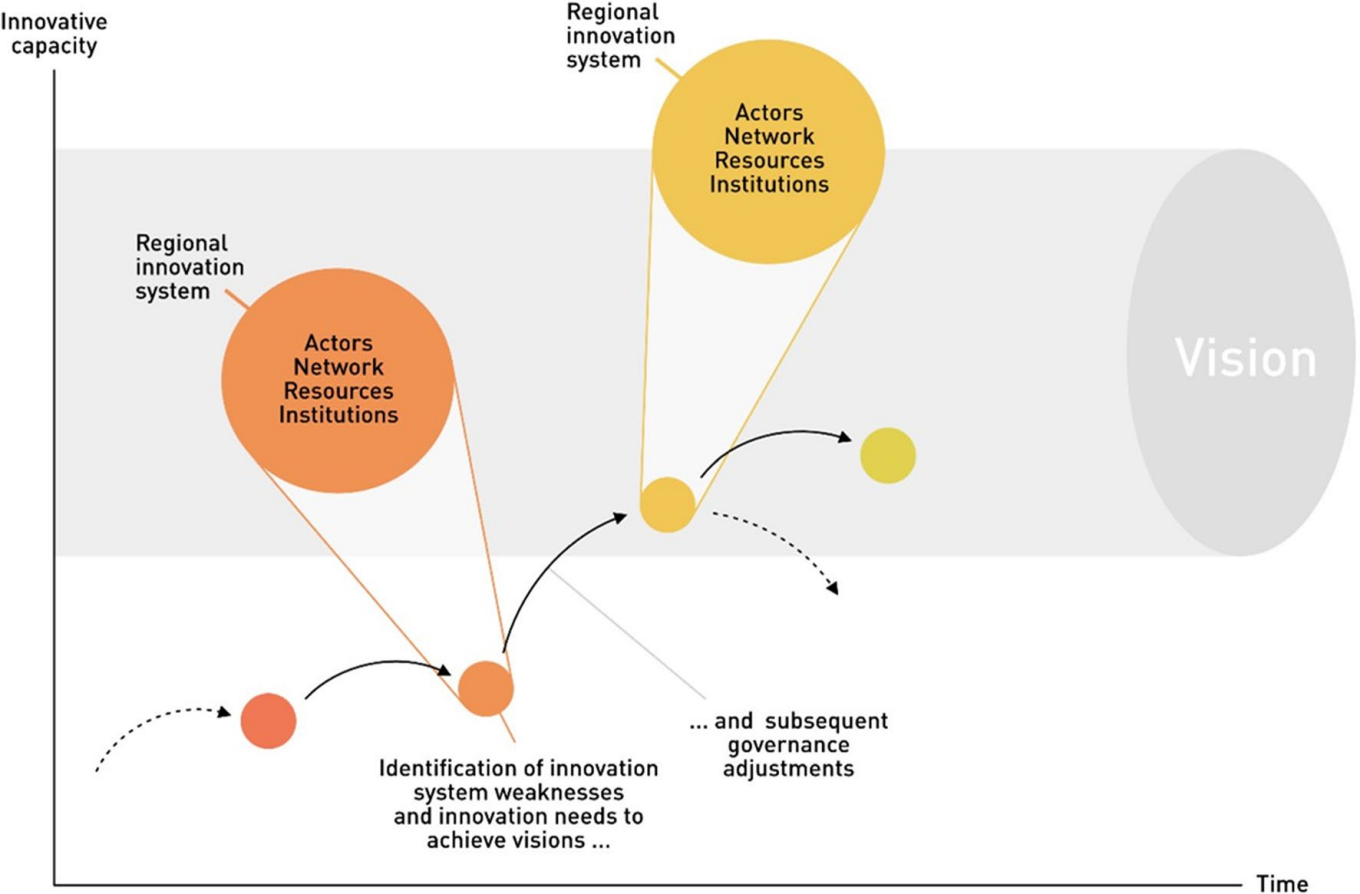
Conceptualisation of how governance adjustments (to RIS structural dimensions evidencing weaknesses) can strengthen innovative capacity, which facilitates achieving visions and overall systemic innovation

Given the importance of understanding innovation system strengths and weaknesses and considering future visions in guiding regional transformations, especially in (peripheral) mountain regions, in this paper, we present an integrated approach to assess and strengthen innovative capacity with respect to the achievement of transformation goals requiring innovation. We use the lens of innovation systems to evaluate the innovative capacity within networks of collaborating actors, and propose how to strengthen such innovative capacity. We use RIS as a mediating concept to study how innovation systems can be harnessed to prepare regional (mountain) actors for systemic innovation (Fig. 1).

## Methods

In this section, we first introduce the two study sites in the Swiss and French Alps, and their system boundaries. We then elaborate our two main methods used, social network analysis (SNA) and visioning workshops. SNA is a method to assess structural capacities and relations between social entities — actors, such as institutions, firms, or individuals — both quantitatively and qualitatively. Since innovative capacities of RIS relate with social interactions of various types, we use SNA. We co-developed visions with stakeholders to have a concrete and normative direction to guide innovation, and accordingly propose network adjustments in line with this direction. Hence, adjustments suggested and discussed in subsequent sections are meant to address both innovation system weaknesses identified in the SNA and the envisioned direction of travel.

While it is not a given that a network will generate innovation, certain network configurations have been seen to be more suitable for generating innovation than others. Using SNA and visioning, we explored system properties (strengths and weaknesses) through the four structural dimensions of innovation systems proposed by Wieczorek and Hekkert (2012): actors, interactions, resources (or infrastructure), and institutions (understood as habits, routines, rules, norms, and strategies). Overall innovative capacity can be interpreted through the analysis of network configurations, as of their structure and composition (e.g. Luthe et al. 2012; Luthe and Wyss 2016). Interactions (at the network level) come through in network structure analyses, while actors and resources are observed in looking at the network’s composition. Institutional problems that could conflict with innovation pathways towards the visions were recorded during the visioning process and are also discussed.

### Study sites

Study site selection responded to our goal of empirically testing our proposition that interventions to strengthen innovative capacities are a particularly important matter in mountain regions that are both geographically and economically peripheral. We therefore selected two complementary study sites in the French and Swiss Alps that would allow comparing innovative capacities between an organisationally thin RIS — Haute-Romanche — and an organisationally thick and specialised RIS — the Visp district. We consider these two sites to be comparable, consisting each of several adjoining mountain municipalities covering areas and spanning altitudes of similar orders of magnitude. Haute-Romanche is located at the far hinterlands of two larger economic regions that extend from an alpine agglomeration (Grenoble) and an urban area with a predominant regional supply function (Gap) (Perlik and Messerli 2004). In contrast, the Visp district is well rooted in a small industrial urban area (Visp-Brig), part of the rather remote Rhone river mountain valley.

#### Haute-Romanche

This region is located in the Central French Alps, covering 204.4 km^2^ at 1300–3900 m.a.s.l. It presents strong constraints from climate, steep terrain, and natural hazards (landslides, avalanches). Population size and density are low, with 800 permanent and 400 temporary residents, and 3.8 people/km^2^. The region is part of the Écrins National Park, which encompasses the municipalities of Villar d’Arêne and La Grave. It only accommodates small- and medium-sized enterprises (SME). La Grave is an international centre for off-piste skiing, climbing, and sightseeing (i.e. niche tourism). The national park receives > 100,000 visitors annually, mostly in summer. One main road connects the region year-round with neighbouring towns for local administration and health services, cultural activities and schooling (40 km away), and with main regional city centres (100 km away). Agriculture concentrates around fifteen livestock farms that produce lamb, beef, and cheese. A more comprehensive study site description can be found in Appendix B.

#### Visp district

The Visp district is located in the Swiss Valais. It covers 443.3 km^2^ at 658–4327 m asl. The region accommodates 15,500 residents. It includes 12 municipalities from the economically growing industrial and urban centre Visp to the touristic destinations in the Saas-valley (Brunner and Grêt-Regamey 2016). Tourism and the leisure industry are major economic factors, although a chemical and biotech international company (Lonza) is the largest employer in the region. Additionally, 161 farms with highly diverse farming activities are active in the region (Grêt-Regamey et al. 2019). A more comprehensive study site description can be found in Appendix B.

### Data collection

#### Social network data

##### Stakeholder identification

We align our stakeholder identification process with the (ecologically sensitive) Quintuple Helix innovation model (Carayannis et al. 2012), which proposes the involvement in the innovation system of actors across academia, industry, government, civil society, and the natural environment to generate knowledge and innovation for system transformations. First, the identification of stakeholders with respect to key challenges being faced by the mountain systems was done through informal exchanges (Haute-Romanche) and semi-structured interviews (Visp district) with key informants. These worked in different sectors (regional planning, agriculture, tourism, natural hazards, environmental conservation, and academia), and some of them had been involved in previous studies (e.g. Lamarque et al. 2011; Brunner and Grêt-Regamey 2016). This information was supplemented by a review of scientific literature and online sources. Information collected for the stakeholders identified included their name, occupation, organisation/s, sector, and scale of action (local to international).

##### Social network structure and composition

Collaboration network data was collected for both sites through an online survey. An example of the questionnaire for Haute-Romanche can be found in Appendix A. The questionnaire was implemented through the online platform ONA Surveys (Optimice 2018), and sent to a cleaned list of 65 key stakeholders in Haute-Romanche and 44 in the Visp district representing all sectors and stakeholder groups previously identified. It presented the question ‘Who do you collaborate with?’, to which respondents could reply by choosing their collaborators from a list including all pre-identified stakeholders (76 in Haute-Romanche, 54 in the Visp district), including organisations and individuals, with the option of adding further actors. For each collaboration, they could also indicate its general purpose (business or private), frequency (seldom (1–2 times/year), monthly, daily-weekly), and whether the interaction had thus far been (perceived as) positive (e.g. good working atmosphere, similar values, and understanding) and/or relevant.

#### Visioning process

Given the need for a shared vision to guide and motivate systemic innovation, we facilitated a process of vision building in both sites. All identified stakeholders were invited to participate in a process of co-construction of visions (e.g. McKee et al. 2015; Longhurst and Chilvers 2019) for the year 2040. These were developed in an iterative process that entailed two consecutive workshops at each site. In Haute-Romanche, stakeholders in groups of 4–5 people were asked to explain how they imagined their region in 2040 in terms of prevailing values, quality of life, and socio-economic activities. Subsequently, two focus groups continued to build on the collective vision. The first one discussed agricultural practices in the region among farmers, while the second brought female stakeholders together to examine habitability. Finally, the collective vision was discussed with 10 targeted stakeholders during semi-structured interviews, to ensure inclusiveness of all relevant stakeholder groups and points of view. In the Visp district, we applied design thinking (Cruickshank and Evans 2012; Chermack and Coons 2015) to structure and stimulate the visioning process (Wiek and Iwaniec 2014), carried out in groups of 3–4 people. The second workshop built on the vision developed in the first one. In it, professional illustrators took visual notes during the moderated design thinking process. Overall, the visioning processes involved 44 individuals in Haute-Romanche and 17 in the Visp district.

### Data analysis

#### Social network structure

Different network configurations are appropriate to address different challenges. Strengths and weaknesses can be assessed by looking at network cohesion, presence of cohesive groups, centralisation, and tie strength (van der Valk et al. 2011a, b; Hur and Park 2016). For instance, fast collective action to solve relatively simple challenges (e.g. response to natural disasters) is more effectively steered in centralised networks (Luthe and Wyss 2016). Nevertheless, the advantage of centrally steering collective action diminishes as problem complexity increases (Berardo and Scholz 2010). Preparing for more complex long-term changes generally benefits from more cohesion and higher modularity, i.e. where clusters incubate new ideas while being connected to a more central, decision-making network core.

Cohesion can be described as the extent to which network actors are connected to each other (van der Valk et al. 2011a, b). This definition spans other concepts like density and connectivity. Innovative capacity changes non-linearly with cohesion. Low cohesion can hinder valuable learning and innovation, a phenomenon known as ‘weak network failure’ (Klein Woolthuis et al. 2005). At the opposite end, ‘overembeddedness’ reduces the susceptibility of the network to novelty (Gilsing et al. 2008; Hur and Park 2016). The cause is a lower diversity of knowledge available and a higher homogenisation of ideas and perceptions, which limits opportunities for novel recombinations (Oh et al. 2004; Gilsing and Duysters 2008).

The presence of (similarly sized) cohesive subgroups has been shown to support the innovative performance of collaboration networks (Hur and Park 2016). Cohesive subgroups are clusters with a larger density of ties between them than to actors in other subnetworks (Boccaletti et al. 2006). The most efficient network architecture for knowledge diffusion is the ‘small world’ topology, which presents cohesive subgroups connected with each other (Cowan and Jonard 2004). This topology allows for intensive knowledge sharing and trust building within subgroups, while permitting the flow of diverse knowledge across the network facilitated by ties between subgroups and relatively short path lengths (Kilduff and Tsai 2003; van der Valk et al. 2011a, b; Ruef 2002).

Networks that have a small group of highly connected actors are often centralised around them, creating ‘hubs’. Peripheral actors with a lower degree of centrality emerge next to these hubs. Higher centralisation often entails a greater ability to prioritise and coordinate activities (Sandström and Carlsson 2008). Additionally, central actors have a clear sense of leadership in the network (Freeman 1978), which is important for innovation as they provide the network with a sense of direction (Rizova 2006; Moolenaar et al. 2010; Moore and Westley 2011). Nonetheless, high centralisation around relatively few actors also presents risks, as it creates a high dependence on their hubs. Their departure from the network would critically affect the structure (van der Valk et al. 2011a, b). Furthermore, Hur and Park (2016) found centralisation to hamper technological diversification, and therefore innovation, due to a limited access of peripheral actors to resources from other parts of the network.

Strong ties between actors facilitate the transmission and assimilation of information. This is especially so across structural holes, where the strength of ties of knowledge brokers to groups to both sides of the hole is critical in guaranteeing the effective flow of knowledge (Hur and Park 2016). Strong ties are also believed to ease the transfer of tacit knowledge (Hansen 1999; Reagans and McEvily 2003). Additionally, there is greater willingness to share the risk of innovation if relationships are trusting, and guided by cooperation rather than self-interest (Uzzi 1997). Nevertheless, weak ties offer greater access to non-redundant information and impose fewer concerns regarding social conformity. This improves opportunities for innovation, as actors connected by strong ties are likely to be more similar and more deferent to each other’s opinions (Hauser et al. 2007; Ruef 2002). Gilsing and Duysters (2008) reconcile these contrasting effects of tie strength by arguing that novelty is created through the mix of strong and weak ties, as strong ones better enable to assess the value of new information and knowledge gathered through weak ties (Table 1).

**Table 1:** Summary of desired visions for 2040 for both study sites

	Haute-Romanche	Visp district
Landscape	Preserved, attractive, and open landscapes supporting local identity, and limited urbanisation	Highly attractive and open landscapes, with regenerative agriculture and balanced old building preservation-renovation
Tourism	Soft four-season tourism focused on natural and cultural local specificities. Open air laboratory with offers of scientific and educational tourism. Strong link with local life and agriculture. Low infrastructure development. Residential tourism and long stays are favoured	Increased offer of high-quality accommodation, and reduction of ‘cold bed’ numbers. Close-to-nature tourism. Mountain railways to consolidate year-round tourism, and connect with sister valley
Agriculture	Local production, processing, and valorisation of agricultural and handicraft products for locals and tourists. Maintenance of traditional mowing and grazing practices on terraces	Strengthened agriculture in close connection with tourism. Climate adapted, small-scale, sustainable farming that meets the regional demand
Settlement	Four season active local life. Preserved and managed villages and hamlets with reduced secondary residents, and developed residential services. Access to digitised work environments for teleworking and co-working. Waste valorised locally and pollution is limited	Preserved and modernised villages. Permanent residents attracted by Industry growth. Flexible digitised work environment for teleworkers
Demography	Population growth within the limits of the region’s capacity and change in community structure (more permanent residents and workers)	Population growth, and change in community structure (more residents and temporary workers)
Forestry	No forestry (no perceived opportunity related to ongoing forest regrowth)	Increased use of local wood to create value-added products and jobs, and promote local identity, green building and landscape preservation
Mobility	Improved accessibility from outside. Favoured car free mobility, soft, shared, and eco-mobility (on foot, by bike, electric bus, carpooling, etc.)	Integrated electric mobility. Controlled protection against natural hazards — 24/7 protection
Energy	Energy self-sufficiency. Glacier-reliant hydropower and solar energy	Glacier-reliant hydropower, wind, and solar energy
Economy	Tourism as the spine of the local economy supporting the development of other sectors. New job opportunities brought by teleworking. Economic performance is not a high priority	Tourism as key economic pillar. Commercially recognised local and regional products
Governance	Cooperation, participation, and consultation for local decisions and economic development	Cooperation and networking between sister valleys for coordinated economic development

Several metrics exist to measure different aspects of the network structure concepts (van der Valk et al. 2011a, b; Hur and Park 2016; Luthe and Wyss 2016). As those metrics are interrelated, there is overlap in the meaning between several of them. We therefore chose the metrics for each concept to maximise complementarity between them and minimise redundancy in the information they provide regarding innovative capacity. Table B1 provides a summary of network structure — and composition (see the ‘Social network composition’ section below) — metrics used, and their interpretation with respect to innovative capacity. R (R Core Team 2018) was used to calculate all metrics, except for the modularity for the core-periphery division (see Table 2), for which we used Ucinet (Borgatti et al. 2002) following the approach presented in Luthe and Wyss (2016).

**Table 2:** Structural characteristics of the two networks. The metrics results presented here relate to their functional interpretation in Table B1

Concept	Metric	Haute-Romanche	Visp district
	Nodes/ties		
	Full network	83/403	64/394
	Core	19/106	21/122
	Periphery	64/38	43/15
Cohesion	Density		
	Full network	0.12	0.19
	Core	0.64	0.48
	Periphery	0.03	0.03
	Average path length	2.21	1.87
	Diameter	5	3
	Global efficiency	0.51	0.59
Presence of cohesive subgroups	Modularity full network	0.22 (4 clusters)	0.17 (4 clusters)
	Modularity core-periphery division	0.67	0.65
	Core-periphery ties/total ties	0.64	0.65
	Clustering coefficient	0.38	0.48
Centralisation Strength of ties	Network centrality (degree)	0.49	0.72
	Degree distribution		
	Skewness	1.89	1.85
	Kurtosis	6.17	6.56
	Frequency: seldom/monthly/daily-weekly	0.43/0.34/0.23	0.41/0.39/0.20
	Relevance: yes/no	0.68/0.32	0.88/0.12
	Positiveness: yes/no	0.54/0.46	0.87/0.13

Network structure metrics, given their broader interpretation with respect to innovative capacity as a whole, reflect what we call the ‘generic’ capacity to innovate of the network. In contrast, we refer to ‘targeted’ innovative capacity as the capacity to generate innovation for overcoming specific envisioned goals by targeting specific network components (Hekkert et al. 2020; Jütting 2020) — measured here by looking at network composition (see ‘Social network composition’ section below).

#### Social network composition

Analysing actor roles is instrumental in understanding systemic weaknesses and adjusting the composition of the network to strengthen its innovative capacity towards the achievement of particular transformation goals.

For collaboration networks to be effective in generating innovation, it is necessary to contemplate network *orchestration* (Durugbo and Lyons 2015; Hölscher et al. 2019). Network orchestration is the monitoring and adjustment of collaborations that is performed by hub organisations or groups of individuals (Dhanaraj and Parkhe 2006). It is generally carried out through board style arrangements embodied by one or several members. Orchestrators play pivotal roles in the creation, evolution, and success of their networks. They can also facilitate the integration and exchange of knowledge and ideas between core and periphery to avoid lock-in effects inherent to very centralised networks (Luthe and Wyss 2016). Nevertheless, orchestrators should not be too dominant and their influence should be complemented with a high density of ties (van der Valk et al. 2011a, b; Hur and Park 2016).

Actors with higher degree centrality scores are better suited for network coordination and steering (Dhanaraj and Parkhe 2006; Binder et al. 2019). Therefore, we propose a selection of potential network orchestrators based on top degree centrality scoring actors. These are selected using Jenks natural breaks classification (Jenks and Caspall 1971). This method optimally allocates values to ‘natural’ classes reducing the variance within classes and maximising the variance between classes. This allows formally identifying potential orchestrators as the actors found in classes where they present more collaboration ties.

Knowledge *brokers* can enhance the organisational potential to generate innovation by bridging structural holes (Hur and Park 2016). The existence of structural holes entails that assorted and non-overlapping knowledge is shared in the network. This knowledge is exchanged efficiently by actors who play the role of knowledge brokers, leading to greater creativity and productivity. Actors who develop ties with disconnected groups or bridge structural holes between neighbours are believed to gain access to a broader range of knowledge than those who are connected to a cohesive one (Granovetter 1973). They are therefore likely to play a major role in generating innovative ideas (Burt 2004; Nerkar and Paruchuri 2005). Given their early access to crucial information, brokers often create new understandings and identify new opportunities that other actors fail to recognise (Burt 2004). Consequently, others perceive them as highly creative.

We identify brokers as articulation points, i.e. nodes that, if removed, increase the number of components in the network (Tian et al. 2017). Brokers can also have a high score of betweenness centrality (Bodin et al. 2006; Everett and Valente 2016). Betweenness measures how much each node helps to minimise the distance between other nodes in the network (Freeman 1978). We therefore also highlight articulation points which have a high score of betweenness centrality.

#### Analysis of visions

We analysed the resulting visions based on audio recordings and illustrations. From these, we derived a vision narrative that brought together the different (non-conflicting) elements of the visions. Where conflicts existed, views with limited support were excluded from the narrative. Ten vision elements were identified in the narratives for both sites: landscape, tourism, agriculture, settlement, demography, forestry, mobility, energy, economy, governance. Given the broad scope of the visions developed, we analysed these elements separately to establish priority focus areas depending on the need for innovation to meet the envisioned changes. In this visioning context, we refer to innovation as new ways of addressing local challenges that are advanced and original. This analysis was carried out in a workshop by the six researchers working in the MntPaths project, which this study is a part of. For each vision element, the degree of innovation required — henceforth, ‘degree of innovativeness’ — was qualitatively discussed and assigned to one of three categories: uninnovative, locally innovative, and globally innovative. An ‘uninnovative’ vision element is one that requires no innovation to address the particular local challenges. A ‘locally innovative’ element means that the proposed ways of addressing local challenges are innovative within the local–regional context, but are not advanced or original beyond the regional level. Finally, a ‘globally innovative’ element signals that proposed ways of addressing local challenges are globally advanced and original, i.e. they are not known to take place anywhere else in mountain areas.

## Results

### Visions 2040

**Fig. 2:**
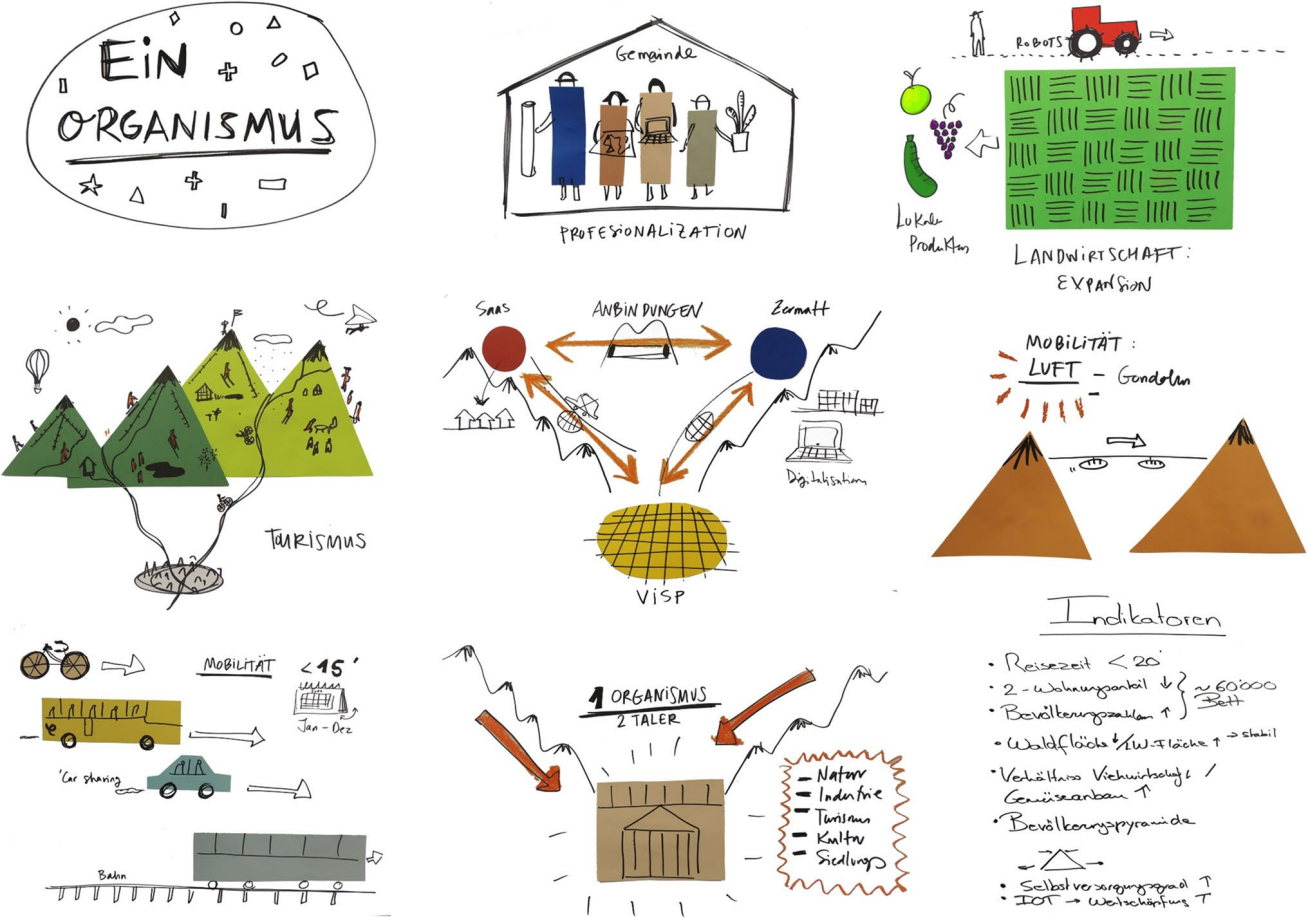
Example of a visual output from a visioning workshop in the Visp district

The visions for both regions highlighted the role of tourism, of sustainable and local value added activities, and of greater cooperation, although with important differences (Table 1). The vision for the Haut-Romanche emphasised an a-growth economic model that embraces tradition and limited infrastructural development. In the Visp district, new technologies play a key role and industry growth is recognised as part of the economic model (Fig. 2). For details, see Appendix C.

It is important to note that, while the visions are largely shared by workshop participants and interviewees, actors in both sites mentioned conflicts, power struggles, and diverging views that likely entail that visions are contested by some (these reflect institutional problems). Such conflicts were more prevalent in Haut-Romanche.

In the following, we describe the vision elements identified as ‘globally innovative’ in each site (see Appendix C for vision innovativeness comparison), as they are the ones requiring the most innovation to be accomplished and therefore the subjects of our targeted innovative capacity analysis.

In Haute-Romanche, we found the elements of the vision dedicated to tourism and the economy to be globally innovative. The vision for tourism involves very limited infrastructure. The tourism offers centres around training and sharing (e.g. high mountain education centre, climate change observation, awareness creation). It seeks a return of contemplative tourism assisted by marked trails. Mountain passes, refuges, and lakes are points of destination and learning. Residential tourism and long stays are favoured. Economic performance is of low importance. A-growth and regional circularity dominate the economic model.

In the Visp district, only the vision for mobility was distinguished as globally innovative. In 2040, the valleys, and especially their access, are 100% protected against natural hazards such as landslides and avalanches around the clock and throughout the year. This security is ensured by the construction and tunnelling of traffic routes, as well as by a new cable car. Areas at risk from natural hazards have been reviewed in zoning plans, and are systematically respected during construction work. Few relocations are necessary. Additionally, the mobility system is fully integrated and electric. Safety is greatly enhanced by autonomous vehicles. The valleys are perfectly connected in 20 min by public transport. There is more cycling infrastructure, and e-bikes make an integral part of the regional mobility offer.

### Social network analysis

Altogether, our analyses of the collaboration network structure indicate that the Visp district seems generally better prepared for innovation than Haute-Romanche; however, we need to differentiate in correspondence with the innovativeness of visions (see below). The Visp district features a faster information flow, a higher capacity to coordinate and steer the network, and higher quality (i.e. relevant and positive) collaboration ties. Survey response rates are presented in Appendix C.

Table 2 shows the network structure metrics for both study sites. The network in the Visp district presents an overall higher cohesion, indicated by a higher density and global efficiency, and a lower average path length and diameter. This network is therefore better prepared for information distribution thanks to a better flow of information, compared to that in Haute-Romanche.

There is some tendency to form cohesive subgroups in both networks. Four clusters can be identified in both, which can facilitate the development of ideas and solutions within the subgroups, and their availability for a potential recombination that may lead to innovation. A clustering coefficient substantially higher than network density also signals the presence of cohesive subgroups throughout both networks. Additionally, the core and periphery form clearly defined modules, and yet there is a large proportion of ties (≥ 64%) dedicated to connecting the core and the periphery. This suggests that a large number of connections in the network can contribute to the influx of new knowledge gathered by peripheral actors to those at the core.

The Visp district presents a substantially more centralised network than Haute-Romanche, which, together with a higher kurtosis (i.e. a more peaked distribution of degree centrality values) and a positive skewness, indicates a higher concentration of connections in the hands of relatively few actors. This reflects, on the one hand, a higher capacity to coordinate and steer the network by few actors towards systemic innovation objectives, presumably through a higher capacity to phase-out path dependency, to embed novelties, and to coordinate orchestration. On the other hand, the higher centralisation can potentially inhibit innovative capacity through limited integration and access of peripheral actors to resources throughout the network, and a greater risk from the potential loss of the hubs — risk that, if materialised, would decrease the potential for social learning, a key element of innovation.

When considering contact frequency, both networks combine strong and weak ties, which should favour a positive balance between the gathering of new knowledge (through weak ties) and the evaluation of its value (through strong ties). However, the network in Haute-Romanche presents a greater proportion of collaboration relationships judged as irrelevant or not positive, which may be reflective of existing conflicts and could erode its potential for collaboration and innovation.

**Fig. 3:**
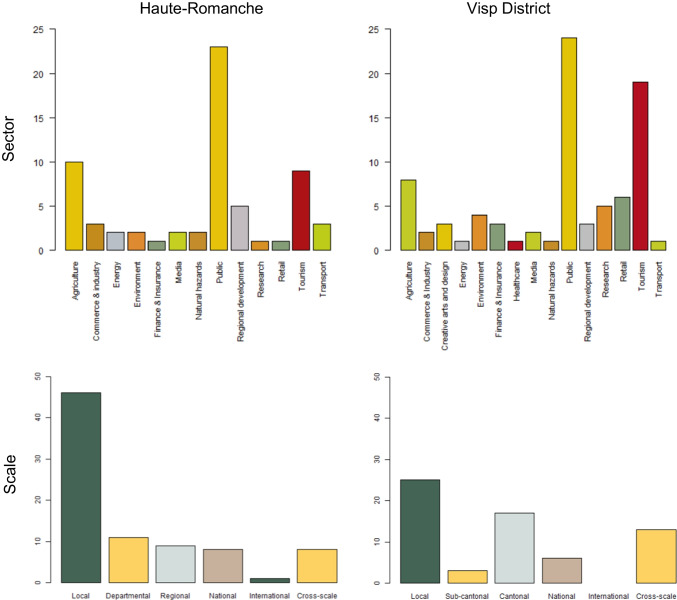
Actor abundance by sector and scale in both study areas

Actor abundance plots (Fig. 3) reflect that, in Haute-Romanche, the sectors contributing most resources to the network are, by far, the public and tourism sectors, followed by agriculture, retail, research, and the environment. All other sectors make a very limited contribution to the network. In the Visp district, the public sector has a much higher representation than all others. The contribution from actors in agriculture, tourism, and regional development is relatively high, compared to that of all other sectors.

Regarding the scale of action of actors, local ones make the largest contribution in both regions, especially in Haute-Romanche. In the Visp district, the resource contribution from the different scales is somewhat more balanced than in Haute-Romanche.

In Haute-Romanche, four actors are identified as potential orchestrators in the two Jenks natural classes with highest degree centrality scores: the deputy major of the municipality of Villar d’Arène and the tourism development society of La Grave (49), the Alpine Station Joseph Fourier (37), a local retail shop (32), and the Laboratory of Alpine Ecology of the University of Grenoble Alpes (31).

**Fig. 4:**
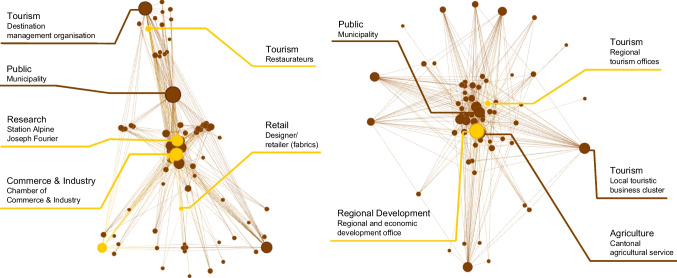
Actor networks in Haute-Romanche (left) and the Visp district (right). Node size indicates the degree centrality of actors, i.e. potential orchestrators. Orange nodes represent ‘cutpoints’, i.e. brokers

In the Visp district, we identified ten potential orchestrators within the two Jenks natural classes with highest degree centrality: the regional and economic development office (56), the cantonal department of agriculture (39), a member of the cantonal Parliament and head of a local cluster of tourism businesses (36), the municipality of Saas-Fee (35), the municipality of Saas-Almagell (33), the chief operating officer of a local cable car company (30), the cantonal natural hazards office (29), the prefect of the Visp district (29), the municipality of Visperterminen (26), and the head of the Visp branch of an engineering company (26). Collaboration networks for both sites are represented in Fig. 4, in which potential orchestrators, as well as brokers, can be distinguished.

Seven actors are identified as articulation points in Haute-Romanche’s network: a local designer and retailer of fabrics, restaurateurs, the press, a departmental Chamber of Commerce and Industry, a departmental office for territorial and rural development, the Station Alpine Joseph Fourier, and a local food retailer. The latter four are also among the 10% of actors with highest betweenness centrality.

In the Visp district, the regional and economic development office and the regional tourism offices act as articulation points, the first one being also the most central actor in the network. Both actors are also among the 10% with highest betweenness centrality scores.

## Discussion

We illustrate how social network structure and composition of RIS can be understood and evaluated to generate innovation that supports mountain system transformations towards desired visions. We show that transformation pathways in the two mountain regions require tailored interventions to strengthen and focus innovation systems due to different innovation endowments and distinct long-term goals towards which to orient innovation. On the one hand, the Visp mountain region shows better preparedness for general innovation given collaboration network characteristics such as a better-defined small world topology, better capacity for coordination, and better quality of collaboration relationships. This may be explained by the efficient and more decentralised Swiss innovation policy, and a rather prosperous, stable economy. On the other hand, the higher centralisation of the Visp district may also have limiting effects on innovation through potentially suppressing innovation from the network’s periphery and clusters that diverges from the goals of the more central actors. The French Haute-Romanche district may have advantages here in allowing — and alluring — a more thriving mental and cultural space for out-of-the-box thinkers to thrive and come up with globally more innovative ideas and visions, as indicated by the comparison of both study site’s visions.

### Requirements and capacities for systemic innovation in mountain regions

According to our analysis, Haute-Romanche is likely to face a greater challenge given the complexity of reshaping economic systems (Renn et al. 1998; Haberl et al. 2011; Ranta et al. 2018; Rosenzweig and Solecki 2018), the novelty of the system envisioned, higher levels of conflict and power struggles, and the structurally lower preparedness of this mountain region for implementing innovation. In contrast, the Visp district is in a relatively better position to achieve its vision through innovation due to lower levels of conflict between regional actors and a higher structural capacity to innovate. However, the resilience of an ‘innovated’ Visp district needs to be seen critically compared to that of the Haute Romanche district: strengthening a business as usual tourism-based mountain economy that relies on high-volume visits and, to a substantial extent, on downhill skiing is a long-term perspective that should be questioned given the clear signs and forecasts of climate change impacts on vanishing glaciers and shortening snow cover season (Hock et al. 2019).

Differences in innovative capacity between the two peripheral regions seem to align with their differently developed RIS. The (thick and specialised) Visp district appears to be better prepared for their type of envisioned innovation thanks to a better information flow, a higher capacity to coordinate and steer the network, and higher quality collaboration ties. Given the importance of policy support in strengthening knowledge attraction and absorption capacities in peripheries (Dawley 2014; Isaksen and Trippl 2016a; Isaksen 2015), the region can also benefit from the prominent role played by public actors in network orchestration, and the internationally connecting company Lonza. Yet, it is important to ensure that mechanisms exist to foster openness to new and different ideas, as centralised steering can lead to lock-in effects if orchestrators (public or otherwise) are too dominant. In Haut-Romanche, the limited quality of relations observed challenges the formalisation of collaborations needed to compensate for the limited knowledge spill-overs in peripheral regions (Grillitsch and Nilsson 2015). The region can, nevertheless, benefit particularly from the involvement of the relatively large number of brokers in the network. However, there is a risk that the non-involvement of some brokers could entail (temporary) breaches in connectivity between parts of the network, hence hindering innovation. To avoid this risk, Haute-Romanche would benefit from strengthening the connectivity across the network, especially where structural holes exist. Notwithstanding, it is important to remember that regional transformations are long-term processes. Hence, constellations of actors involved in them should be somewhat adaptable throughout in response to reflexive adjustments in visions and the articulation of best practices (Loorbach 2010; Wittmayer et al. 2017). A question here is how interdependent the network and the shared vision are, and how they influence each other. In Visp, the question arises, if the network structure only supports a status-quo economy and dominant ideas to thrive and develop. In Haute Romanche, it is questionable if the network structure and composition will allow for more alternative and more substantially different ideas to thrive, or if it is the other way around where the economic situation nudges people to develop different ideas, which are visible in the network structure.

The collaboration between actors across scales, from the local to the national level, in both sites (especially in the Visp district) provides flexibility and diversity, supporting social learning and innovation (Cash et al. 2003; Luthe and Wyss 2016). These capacities are advantageous to the governance of resilience and sustainability interventions in such regions (Lebel et al. 2006; Patterson et al. 2017). Nonetheless, the lack of collaboration ties with actors from other countries or operating internationally is an important system weakness (Isaksen and Trippl 2016b; Eder and Trippl 2019) affecting the capacity to innovate in both regions, especially in Haute-Romanche. The Visp district’s thicker and more specialised RIS makes it less vulnerable to this potential weakness. Transnational collaboration has been shown to provide access to resources that can help stimulate sustainability-oriented innovation (Wieczorek et al. 2015; Wieczorek 2018). Transnational networks can be very effective in promoting the exchange of non-redundant, often context-specific, knowledge that support innovation in regions, cities, and organisations (Lange and Buttner 2010; de Noni et al. 2018; Lee 2018). This can make the difference in achieving systemic innovation in peripheral regions, like Haute-Romanche.

The dominance in both sites of local and regional actors among orchestrators and brokers points to ‘grassroots’ RIS, i.e. RIS where policy and business governance are mainly organised at the local–regional level (Cooke 2004; Stuck et al. 2016). This seems to signal a misalignment with their national-regional innovation policies. The strong national and cantonal innovation policy in the Visp district might have hinted at a ‘networked’ RIS, i.e. a RIS that involves a multilevel approach to governance. This discrepancy may be explained by the strong decentralisation of decision-making in Switzerland and/or by the peripheral nature of the district, even within the canton. Similarly, a grassroots RIS in Haut-Romanche is likely an emergent outcome of the legacy of the former ‘dirigiste’ (i.e. nationally orchestrated) innovation policy that supported the region’s touristic development, and a regional innovation policy with little influence in the more peripheral mountainous areas.

Identifying mountain regions as peripheral, grassroots RIS is useful for understanding their potential for systemic innovation. Led by local actors, grassroots movements have been shown to support innovation that address some of the envisioned goals pursued in the studied mountain regions, such as energy autarky, sustainable agriculture, or an economic system that prioritises qualitative over quantitative growth (Hossain 2016) (Hossain 2018). While scaling innovations is a main challenge to grassroots and peripheral RIS, these types of RIS can be sufficient to generate small-scale innovations that support the goals of local and regional mountain communities.

### Guiding innovation towards desired system transformations

Assessing the targeted capacity to innovate by looking at network components can contribute to adaptive governance by understanding the adjustments required to strengthen communities towards the achievement of shared visions (or vision elements) through innovation. This can be illustrated through the example of the globally innovative vision elements in Haute-Romanche: tourism and the a-growth economy. Our analysis of the resources that actors contribute to the network reveals that the public and tourism sectors have a high representation in it. This largely reflects the important role of these sectors in the development of the region. For a community aiming at redefining its tourism-based economy, there is an obvious need to fully involve the public and tourism sectors in the process of systemic innovation. However, the overrepresentation of these sectors entails that overlapping resources are supplied to the network, creating redundancy and competition that can hamper innovation (Burt 1997; van der Valk et al. 2011a, b). Conversely, the underrepresentation of several important sectors can limit the amount and diversity of sector-specific information that could contribute towards the vision. A greater involvement of actors dealing with natural hazards and insurance, in the creative arts and design industry, or the media can make a substantial contribution towards the type of low-intensity contemplative and interpretive tourism envisioned (Müller and Weber 2008; Richards 2011; Healy et al. 2016). Analogously, innovation that targets regional circularity of the economy requires greater collaboration with a broader variety of economic and societal stakeholders (e.g. in commerce, industry, transport, regional development) to enable the circular flow of materials and related efficiency benefits (Geng et al. 2012; Kalmykova et al. 2018; OECD 2019). Importantly, conflicts between municipalities, and between the national park and entrepreneurs, can handicap the transformation process (for an in-depth assessment of conflicts in both regions, see Adler et al. 2022). Nevertheless, acknowledging them and working towards their resolution could be an important step towards the achievement of both vision elements.

The literature calls for a shift in innovation policy that supports transformative change directed towards addressing contemporary challenges (Schot and Steinmueller 2018; Tödtling and Trippl 2018). New policies should focus on directionality of socio-technical systems and more inclusive and participatory approaches (Levy and Lubell 2018). Weber and Rohracher (2012) suggest that, to support goal-oriented transformative change, policies need to address four transformational system failures: directionality, policy coordination, demand-articulation, and reflexivity. The approach presented here is helpful in starting to address some of them. It provides, through the visioning process, an agreed direction to guide collaborative efforts, and reflect on necessary governance changes, for systemic innovation. Additionally, understanding existing collaboration networks and their alignment (or lack thereof) with shared goals helps in addressing cross-industry and multi-level policy coordination failures. The analysis can inform the incorporation of coordination improvements during the construction of transformative change pathways (Schot and Steinmueller 2018).

Our approach could be particularly valuable in strengthening RIS to support resilience building and sustainable development in mountain regions. It can help in identifying and promoting valuable collaboration relationships in regions that lack the frequent spontaneous interactions that support innovation in core regions. As our analysis has shown, regional innovation policy may not fully benefit mountain regions due to their peripherality. The presented approach is helpful in flagging such situations and can help in creating the necessary bridges to start addressing this imbalance in regional innovation endowment. To operationalise this, an approach as presented here should be integrated within existing regional innovation policy frameworks. For instance, our approach could provide a conceptual and analytical basis to guide RIS ‘smart specialisation strategies’ that support greater regional sustainability. Smart specialisation is a place-based approach to regional development characterised by the identification of strategic areas for intervention with wide stakeholder involvement (Foray 2015; Foray et al. 2018). Our approach could help in establishing the information basis to monitor and adjust the multi-stakeholder governance that sustains the entrepreneurial discovery process (Marinelli and Perianez-Forte 2017; Mariussen et al. 2019).

### Limitations and future research

We only illustrate network properties and required changes at one point in time. However, the governance of systemic innovation needs to be adaptive in order to overcome new challenges arising over time (Folke et al. 2005; Ojha et al. 2012; Cumming et al. 2013; Lee and Petts 2013). This raises the question of what organisational set-up(s) allow effectively orchestrating systemic innovation, while remaining active and adaptive over long time periods.

A potential empirical limitation of our study is the assumption that a vision developed by a number of ‘representative’ stakeholders is shared, and therefore pursued, by all collaboration network actors. The number of actors involved in the visioning process in the Visp district (17) could raise questions about the representativeness of their vision for a total population of 15500 inhabitants. However, the representation across diverse actor types and economic sectors in the process does contribute towards a broad representativity of the vision.

Furthermore, network analysis results should be understood in the light of limited data availability. Even though our studies’ survey response rates were moderate and high in the respective sites, important stakeholders could still be missing from our sample due to self-selection. This is, nevertheless, commonplace to studies using social network data. As well, studies confirm that survey respondents are often those with more connections in a network; higher response rates with more peripheral actors would thus probably not substantially change the analysis (Levy and Lubell 2018).

Nevertheless, the absence of the company Lonza from the SNA results challenges the system boundaries of the Visp RIS as reflected in such results. This company makes the Visp RIS globally connected, and provides economic stimuli through diversification beyond tourism. Due to these characteristics, the addition of the network of a Lonza representative might have enriched our analyses. However, the lack of response to the SNA survey from Lonza surveyees led to the absence of the company and its network from the SNA.

Finally, our assessment of access to resources only considers sectoral and scale-specific contributions. However, studies looking at the development of innovative solutions to achieve particular vision elements would benefit from a more nuanced understanding of resources. For instance, information on the amount and type of resources (e.g. human, physical, financial, natural, digital) provided by network actors could help identify resource problems, as well as the potential contribution of actors to the resolution of concrete challenges.

## Conclusions

Mountain regions, particularly economically and geographically peripheral ones, can benefit from strengthening their capacity to innovate in ways that support the achievement of socially desired visions. Analysing and comparing network structures allows to understand the generic innovative capacity of the network. Looking into network components allows to identify and tackle actor and resource weaknesses in regional innovative capacities for addressing envisioned goals.

Strengthening RIS capacity for systemic innovation requires better representativeness across sectors that is appropriate to meet envisioned challenges, and the creation of meaningful transnational mountain collaboration relationships. We therefore propose the development of transnational mountain collaboration networks geared towards supporting resilience, sustainability, or other overarching goals wished for by mountain communities. Such a governance innovation would allow mountain regions to share (tacit) knowledge and ideas, and learn about experiments and successful and failed interventions, as well as to build legitimacy and pool resources, in support of systemic innovation. The identification of orchestrators is also important, as they can play a key role in coordinating the re-design of their networks to govern systemic innovation effectively. Integrating conceptual and empirical approaches as presented here into regional innovation policy instruments can help in understanding and strengthening collaboration networks to support system transformations in mountains.

## Supplementary Information

Below is the link to the electronic supplementary material.

Supplementary file1 (PDF 604 KB)

Supplementary file2 (PDF 171 KB)

Supplementary file3 (PDF 1435 KB)
