# Supplementary material for: Aligning social networks and co-designed visions to foster systemic innovation in the Alps
Source: Reg Environ Change. 2023 Jul 28;23(3):102. doi: 10.1007/s10113-023-02099-y (PMC10382410; doi:10.1007/s10113-023-02099-y)
Supplement: Supplementary file 1 — Supplementary file1 (PDF 604 KB) [file 10113_2023_2099_MOESM1_ESM.pdf]

Appendix A

Collaboration questionnaire

Thank you for agreeing to take part in this important survey looking at your collaboration with other actors as part of your work. It will help us (and you, in a coming workshop) to understand the existing and potential for collaboration between actors relevant to the development of future pathways of adaptation in the Lautaret. For clarification, by collaboration we mean sharing information, advice or resources of any kind, as well as providing or receiving services that help you do your work. Examples can be a public institution giving you a mandate, discussions and negotiations, you providing advice to someone, or an organisation offering you economic support for your business activities.

This survey should only take 15-20 minutes to complete. Be assured that we will keep all answers you provide in the strictest confidentiality.

Name: Organisation: Function:

**Stakeholder**	**Who do you collaborate with?**		**What is the aim of collaboration with this actor?**	**How regularly do you collaborate?**			**Has this collaboration been of relevance for your organisation?**	**Has this collaboration been positive (e.g. good working atmosphere, same values and understanding)?**	**Does your organisation intend to intensify this cooperation in the future?**		
	Business	Private	(Insérez la lettre)<br>A. Diversification de l'économie.<br>B. Développement du tourisme.<br>C. Maintien/ augmentation du revenu des agriculteurs.<br>D. Distribution des propriétés agricoles.<br>Autres (lequel/s?)<br>E. ……………………….<br>F. ……………………….<br>G. ……………………….	Seldom (1-2 times/ year)	Monthly	Daily-Weekly	(If yes, please indicate with an X)	(If yes, please indicate with an X)	Yes	No	Maybe
**ADDET (Agence départementale de développement économique et touristique des Hautes-Alpes)**											
**AFP (Association Foncière Pastorale)**											
**Agence Régionale de Santé**											
**Agriculteurs**											
**Armée**											
**ASA (Associations Syndicales Autorisées)**											
**Association défense de l'environment**											
**Association de commerçants de La Grave**											
**Association de commerçants de Villar d'Arêne**											
**Association de conseil aux entreprises**											
**Associations des commerçants**											
**Associations pour le mantien d'une agriculture paysanne**											
**Assurances**											
**Banque**											
**Bureau des guides**											
**CDOA (Commission Départementale d'Orientation Agricole)**											
**CERPAM**											
**Chambre d'Agriculture**											
**Chambre de Commerce et de l'industrie**											
**Chambres consulaires**											
**Comcom Briançonais**											
**ComCom Oisans**											
**Commerçants de la Guisane/ Remontées mécaniques**											
**Commerçants du Briançonais**											
**Conseil de développement**											
**Cooperatives laitieres/ Fruitières**											
**DDT (l'État)**											
**Département de l'Isère**											
**Département des Hautes-Alpes**											
**Député des Hautes Alpes**											
**Députés (de l'Isère, des Haute's Alpes, de l'ANEM)**											
**DGFIP-URSAAF**											
**Ecole du Ski Français**											
**État législateur**											
**FROTSI/ Trajectoires Tourisme**											
**GIRN Alpes**											
**Grosses stations ski (Alpes d'Huez/Deux Alpes/ Serres Chevalier)**											

**Groupe d'action local**											
**Habitants travaillant dans le tourisme**											
**Hébergeurs**											
**Initiative Nord Hautes-Alpes**											
**L'État**											
**Mairie de La Grave**											
**Mairie de Villar d'Arêne**											
**Autres mairies du Briançonnais**											
**Autres mairies de l'Oisans**											
**Médiateur crédit**											
**TPE/PME**											
**Office du tourisme de La Grave**											
**Office du tourisme du Bourg d'Oisins**											
**Parc national des Écrins**											
**PARN (Pôle Alpin Risques Naturels)**											
**Pays du Grand Briançonnais**											
**Pépinière d'entreprises**											
**Pierre Leroy (Pays du Grand Briançonnais)**											
**Poste**											
**Préfecture**											
**Presse**											
**Propriétaires fonciers**											
**Région**											
**Remontées mécaniques**											
**Restaurateurs**											
**Restauration des Terrains en Montagne de l'ONF**											
**RSI**											
**SAFER**											
**SATA (Société d'Aménagement Touristique Alpe d'Huez)**											
**Scientifiques**											
**Sécurité civile/ Commission montagne**											
**SEM citoyenne de prod d'energie**											
**Stations de ski**											
**Syndicats agricoles**											
**Touristes/Visiteurs**											
**UE**											
**.................................**											
**.................................**											
**.................................**											
**.................................**											

*Catégories:
A. Diversification de l'économie
B. Développement du tourisme
C. Maintien/ augmentation du revenu des agriculteurs
D. Distribution des propriétés agricoles
Autres (lequel/s?)
E. ……………………….
F. ……………………….
G. ……………………….

From the actors you do not collaborate with, who would you like to collaborate in the future? Please, circle relevant actors in the left column of the table.
