# Supplementary material for: Aligning social networks and co-designed visions to foster systemic innovation in the Alps
Source: Reg Environ Change. 2023 Jul 28;23(3):102. doi: 10.1007/s10113-023-02099-y (PMC10382410; doi:10.1007/s10113-023-02099-y)
Supplement: Supplementary file 2 — Supplementary file2 (PDF 171 KB) [file 10113_2023_2099_MOESM2_ESM.pdf]

Appendix B

Complete study site descriptions

Haute-Romanche

This region is located in the Central French Alps, covering 45 km² at 1300-3900 m.a.s.l. It presents strong constraints from climate, steep terrain and natural hazards (landslides, avalanches). Population size and density are low, with 800 permanent and 400 temporary residents, and 3.8 people/km^2. The region is part of the Écrins National Park, which encompasses the municipalities of Villar d'Arêne and La Grave. It only accommodates small and medium-sized enterprises (SME). La Grave is an international centre for off-piste skiing, climbing and sightseeing (i.e., niche tourism). The national park receives >100,000 visitors annually, mostly in summer. One main road connects the region year-round with neighbouring towns for local administration and health services, cultural activities and schooling (40 km away), and with main regional city centres (100 km away). Agriculture concentrates around fifteen livestock farms that produce lamb, beef and cheese.

The site sits at the periphery of a regional cluster of (mainstream) large skiing resorts, whose planning and development was largely orchestrated from the national level in 1960-1980 (George-Marcelpoil and François 2012). Innovation policy has been gradually decentralised over the past 15 years (Harfi et al. 2016). Today, it consists mainly of nationally administered R&D tax incentives and advisory services, and regionally managed EU innovation subsidies.

Tourism demand has nevertheless decreased in recent years. Mean annual temperature has increased by 1 °C over the last 30 years and precipitation is more variable (Nettier et al. 2017). Reduced snowfall challenges winter activities, although La Grave holds a competitive advantage over resorts at lower altitudes. High altitude mountaineering activities are also vulnerable to glacier melt, growing rockfall risk and more frequent heat waves. Furthermore, growing interannual climate variability and increased drought risk pose important challenges to the persistence of the current farming system (Lamarque et al. 2013).

Local governance is largely via the municipalities, and the mountain guides and shopkeepers' associations. The farming system is largely dominated by collective institutions that decide, e.g., on land allocation or regional market development. Farmers are dependent on agri-environmental EU subsidies (up to 80% of farm income) and on tourism for off-farm employment and real estate income (Schermer et al. 2016). Furthermore, tourism is harnessed through national park regulations and assisted by publicprivate investments and subsidies. Property development is regulated by strict planning rules and limited to the villages. Further information on this site can be found in Lavorel et al. (2019).

Visp district

The Visp district is located in the Swiss Valais. It covers 443.3 km^2 at 658-4327m asl. The region accommodates 15,500 residents. It includes 12 municipalities from the economically growing industrial and urban centre Visp to the touristic destinations in the Saas-valley (Brunner and Grêt-Regamey 2016). Tourism and the leisure industry are major economic factors, although a chemical and biotech international company (Lonza) is the largest employer in the region. Additionally, 161 farms with highly diverse farming activities are active in the region (Grêt-Regamey et al. 2019).

Swiss innovation policy understands the generation of innovation as a core task of industry and SME (SwissCore 2021). The state supports innovation by providing funding (mainly to publicly-oriented research institutes and universities), various advisory services, and networks, yet without dictating technology or sector-specific investments in innovation. Swiss cantons offer their own innovation promotion initiatives. Valais provides advisory services, support for higher education, training and research institutions, and facilitates sectoral diversification and inter-sectoral synergies (Canton du Valais 2012). The canton hosts a distributed technopark with a focus on ICT, life sciences and renewable energies (ca. 40-100 km away from the Visp district), including a regional university of applied sciences and a branch of a Swiss technical university (EPFL). A regional and economic development agency is the main actor promoting collaboration across boundaries and sectors in the Visp district.

The region is among the driest in the Swiss Alps, and further decline in precipitation is expected, as well as a rise of mean temperature (Huber et al. 2013). Several natural hazards affect this region regularly, avalanches being the most frequent, and their likelihood is expected to increase with climate change (Brand et al. 2013). These threaten tourism through impacts on infrastructure and a decrease in visitor numbers due to fear of natural hazards (Nöthiger and Elsasser 2004). Additionally, snow reliability, which is crucial for winter tourism, will change with climatic change (Rixen et al. 2011).

Farms are highly dependent on subsidies (over 50% of farmer income) and over 90% of them are operated part-time. Farm income is mainly supplemented by that generated from tourism activities. Forests (20% of land cover) are maintained to protect against avalanches and other hazards. Federal forest and spatial planning regulations constrain land use in mountains through the prioritisation of forest conservation and the designation of building zones, respectively. The percentage of secondary homes in each municipality is restricted to 20% or lower to prevent urban sprawl. For further information on this site see Brand et al. (2013) and Brunner and Grêt-Regamey (2016).

References

Brand FS, Seidl R, Le QB, et al (2013) Constructing Consistent Multiscale Scenarios by Transdisciplinary Processes: the Case of Mountain Regions Facing Global Change. Ecology and Society 18:. https://doi.org/10.5751/ES-04972-180243

Brunner SH, Grêt-Regamey A (2016) Policy strategies to foster the resilience of mountain socialecological systems under uncertain global change. Environmental Science & Policy 66:129–139. https://doi.org/10.1016/j.envsci.2016.09.003

Canton du Valais (2012) Stratégie de développement économique du Conseil d'Etat. Sion, Switzerland

Freeman LC (1978) Centrality in social networks conceptual clarification. Social Networks 1:215–239. https://doi.org/https://doi.org/10.1016/0378-8733(78)90021-7

George-Marcelpoil E, François H (2012) De la construction à la gestion des stations. Journal of Alpine Research. https://doi.org/10.4000/rga.1897

Grêt-Regamey A, Huber SH, Huber R (2019) Actors' diversity and the resilience of social-ecological systems to global change. Nature Sustainability. https://doi.org/10.1038/s41893-019-0236-z

Harfi M, Lallement R, Pisani-Ferry J (2016) Quinze ans de politiques d'innovation en France, rapport de la Commission nationale d'évaluation des politiques d'innovation. Paris

Huber R, Rigling A, Bebi P, et al (2013) Sustainable Land Use in Mountain Regions Under Global Change: Synthesis Across Scales and Disciplines. Ecology and Society 18:. https://doi.org/10.5751/ES-05499-180336

Lamarque P, Artaux A, Barnaud C, et al (2013) Taking into account farmers' decision making to map fine-scale land management adaptation to climate and socio-economic scenarios. Landscape and Urban Planning 119:147–157. https://doi.org/10.1016/j.landurbplan.2013.07.012

Lavorel S, Colloff MJ, Locatelli B, et al (2019) Mustering the power of ecosystems for adaptation to climate change. Environmental Science & Policy 92:87–97. https://doi.org/10.1016/j.envsci.2018.11.010

Nettier B, Dobremez L, Lavorel S, Brunschwig G (2017) Resilience as a framework for analyzing the adaptation of mountain summer pasture systems to climate change. Ecology and Society 22:. https://doi.org/10.5751/ES-09625-220425

Nöthiger C, Elsasser H (2004) Natural Hazards and Tourism: New Findings on the European Alps. Mountain Research and Development 24:4,24-27

Rixen C, Teich M, Lardelli C, et al (2011) Winter Tourism and Climate Change in the Alps: An Assessment of Resource Consumption, Snow Reliability, and Future Snowmaking Potential. Mountain Research and Development 31:8,229-236

Schermer M, Darnhofer I, Daugstad K, et al (2016) Institutional impacts on the resilience of mountain grasslands: an analysis based on three European case studies. Land Use Policy 52:382–391. https://doi.org/10.1016/j.landusepol.2015.12.009

SwissCore (2021) Swiss innovation system. https://www.swisscore.org/swiss-knowledge/innovation. Accessed 10 Feb 2021

Table B1. Framework to assess generic and targeted innovative capacites based on innovation systems structural elements and network concepts and metrics.

Innovative capacity evaluation type	Innovation system structural dimension	Concept	Metric	Description	Range of values	Meaning for innovative capacity
Generic	Interactions	Cohesion	Density	Ratio between the existing number of ties and the maximum possible number of ties.	[0,1]	Higher density leads to greater trust, but can also conduce to homogenisation of ideas and perceptions.
			Average path length	Average length of all paths between all nodes in the network.	> 0	Shorter path length suggests overall faster information flow. Important to keep cohesive subgroups interconnected.
			Diameter	Size of the longest possible path between two nodes in the network.	> 0	Indicates the effectiveness of the network in connecting pairs of potential collaborators.
			Global efficiency *	Measure of the capability of a network to exchange information. Mean of the inverse tie distances on a network level.	(0,1]	Higher value indicates higherer efficiency in the exchange of information.
		Presence of cohesive subgroups	Modularity index	Measure for the tendency to form modular subgroups in a graph, where a group of nodes has more dense ties within than to nodes outside the group. Fraction of the ties that fall within the given subgroups minus the expected fraction if ties were distributed at random.	[-0.5,1]	Positive, and preferably larger, modularity values reflect the presence of subgroups. Their presence supports diversity in knowledge and ideas, which may lead to innovation as these are shared across subgroups and recombined.
			Prevalence of core-periphery ties	Ratio between the ties connecting the core and periphery of the network, and the total number of ties.	(0,1]	Higher prevalence provides better support for the flow of ideas between the core and the periphery.
			Clustering coefficient	Mean of the clustering coefficient of all nodes. For a node, it is the ratio between the number of ties connecting its neighbourhood and the total possible number of ties in that neighbourhood. Cohesive subgroups are present, if it is higher than network density.	[0,1]	Higher clustering coefficient points to the existence of strong local knowledge flows. It can enable innovation in the presence of a short average path length and diameter.
		Centralisation	Network degree centrality	The degree of inequality or variance in the network as a percentage of that of a perfect star network of the same size.	[0,1]	High network centrality supports leadership and coordination in the network, but may limit the participation of less central actors in social learning.
			Degree distribution	Skewness and kurtosis of the distribution of degrees.	No limited range	Higher positive skewness and a leptokurtic (i.e., highly peaked) degree distribution shows that the hubs exist among a large number of more limitedly connected others. A network with relatively few hubs can be easier for them to lead and coordinate, but may suffer if they exit it.
		Strength of ties	Frequency	Proportion of the total number of ties that represent seldom (1-2 times a year), monthly and daily-weekly collaborations.	[0,1]	Higher frequency entails greater trust and willingness to share risks, while lower frequency facilitates access to non-redundant information and reduces the pressure for social conformity. Innovation is best enabled through a

						balance between frequent and infrequent collaborations.
			Relevance	Proportion of the total number of ties that represent collaborations perceived as relevant, or not, with respect to collaboration objectives.	[0,1]	The legacy of irrelevant collaborations may hinder the willingness of actors to become involved in new collaborations for innovation.
			Positiveness	Proportion of the total number of ties that represent collaborations perceived as positive.	[0,1]	Higher positiveness of collaborations reflect higher chances that actors will be willing to share the risk of innovation.
Targeted	Resources/ actors	Access to resources across sectors	Actor presence and abundance by sector	Sectors present in the network, and number of actors belonging to each of them.	No limited range	Greater access to resources from sectors relevant to the specific vision elements targeted (esp. those requiring most innovation) increases strategic fit and resource complementarity.
		Access to resources across scales	Actor presence and abundance by scale	Scales of action of actors present in the network, and number of actors belonging to each of them.	No limited range	Greater access to resources from actors operating at scales relevant to the specific vision elements targeted (esp. those requiring most innovation) increases strategic fit and resource complementarity. The presence of actors collaborating internationally provides global knowledge linkages that increase innovative capacity.
	Actors	Orchestration	Node degree centrality	Potential orchestrators are identified based on top centrality (i.e., number of ties of each node) scores.	No limited range	Orchestrators play key roles in monitoring and adjusting collaborations, and can facilitate knowledge integration and exchange.
		Knowledge brokerage	Articulation points	Nodes that, if removed, increase the number of components in the network.	{0,1}	Brokers develop ties with disconnected groups, which confers opportunities to access different streams of knowledge. Hence, they create new understandings and identify new opportunities.
			Node betweenness centrality	Percentage of shortest paths that must go through each node (considered only for articulation points).	[0,1]	Brokers bridge structural holes. This confers opportunities to access different streams of knowledge. Hence, they create new understandings and identify new opportunities.
	Institutions	Institutional problems	Presence of institutions	Presence/absence of habits, routines, rules, norms and strategies.	-	Absent institutions may inhibit innovation.
			Capacity of institutions	Stringency/weakness of habits, routines, rules, norms and strategies.	-	Stringent institutions may lead to the so-called appropriability trap and favour incumbents.<br>Weak institutions may curb innovation, e.g. by insufficiently supporting new developments.

* While this metric contributes very similar information to average path length (and we would therefore rather remove it to avoid redundancy) we include it to allow for comparison with previous studies that have used this metric.
