# Supplementary material for: Aligning social networks and co-designed visions to foster systemic innovation in the Alps
Source: Reg Environ Change. 2023 Jul 28;23(3):102. doi: 10.1007/s10113-023-02099-y (PMC10382410; doi:10.1007/s10113-023-02099-y)
Supplement: Supplementary file 3 — Supplementary file3 (PDF 1435 KB) [file 10113_2023_2099_MOESM3_ESM.pdf]

Appendix C

Survey results

Survey results are shown in Table C.1. Response rates were moderate in Haute-Romanche and high in the Visp district, compared to those observed in previously published surveys of collaboration networks (e.g. Kelman et al., 2016; Luthe et al., 2012). Response rates by sector and scale of action are shown in Figure C.1. Response rates per sector were moderate to high. Surveyed sectors that did not participate were natural hazards and healthcare in Haute-Romanche, and forestry in the Visp district (with one surveyee per sector). Response rates by scale were moderate to high. No national or international actors were surveyed in Haute-Romanche, while no international or cross-scale actors (i.e., those acting at different scales) were surveyed in the Visp district (as no individuals were identified for stakeholders in those categories).

Table C.1. Response statistics.

	Haute-Romanche	Visp district
**Surveyees**	65	44
**Respondents (response rate)**	24 (37%)	25 (57%)
**Completed (completion rate)**	22 (34%)	22(50%)

Figure C.1. Response rates by sector and scale for Haut-Romanche and the Visp district.

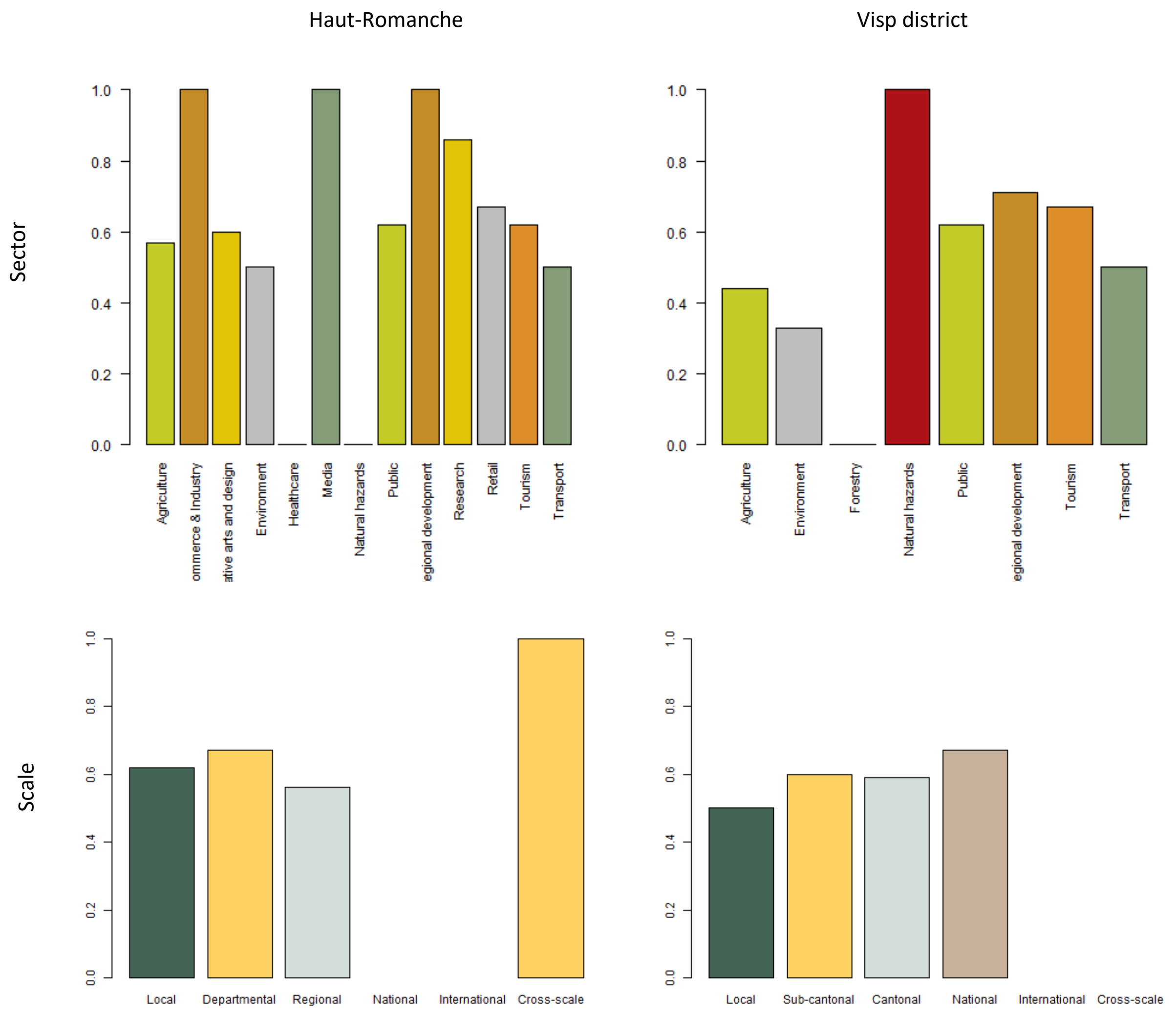

Visions for Haut-Romanche and the Visp District

	Haut-Romanche	Visp district
Landscape	The landscape is preserved and attractive.<br>Landscape supports the local identity and legacies.<br>Landscape is maintained open by regenerative agriculture.<br>Urban sprawl remains marginal. No large infrastructures.	Very high attractiveness of the landscape in 2040:<br>- Kept open with healthy, small-scale, regenerative agriculture.<br>- Respectful renovation of old buildings and conversion of old stables into small apartments.
Tourism	The tourist offer extends over the four seasons, supported by all tourism professionals.<br>A soft and flexible tourism is proposed based on local natural and cultural specificities.<br>The region is seen as an open-air laboratory to develop new mountain practices and offers of scientific and educational tourism linked to climate change.<br>No large infrastructure development linked to tourism.<br>Residential tourism and long stays are favoured, as service supply is sufficient.<br>The winter off-piste ski identity is maintained.<br>Tourism benefits from strong links to local life and agriculture.	Considerable increase in offer of high quality accommodation. Even for spontaneous travellers, freelancers and young people on a reduced budget, attractive rooms and apartments are available in sufficient numbers. New concepts of multiple uses led to fewer empty dwellings and the number of cold beds is limited to ca. 60,000. Tourism is a pillar of the regional economy. The development of a close to nature and sustainable tourism was achieved by making tourism a school subject. The resulting better understanding of tourism workers has made the valleys one of the most attractive tourist regions of the Alps. The strong diversification and internationalization of seasonal workers also contributed to this.<br>Mountain railways are consolidated:<br>- Fewer mountain railways in the valley.<br>- Mountain railways focused on a few, well-used and economically healthy ascent aids that support year-round tourism.<br>- Zermatt and Saas Fee are directly connected, which is possible thanks to two new cable cars.
Agriculture	Diversification of agricultural and artisanal products based on natural and local resources.<br>Maintenance of traditional mowing and grazing practices on terraces.<br>Supported by local and regional social and economic networks for transformation, valorisation and distribution of products.<br>Agriculture is strongly linked to tourism for the sharing and discovery of local “savoir-faire”.<br>Agriculture strongly contributes to landscape attractiveness, and respects the environment and natural resources.	Strengthening agriculture in close connection with tourism:<br>- Regional agriculture has developed and can supply the valley seasonally.<br>- Growth is mainly qualitative because of small scale farming methods adapted to climate change.<br>-Agriculture is diversified and closely linked to natural and experiential tourism. Traditional farming and processing and rearing methods are part of such tourist experiences.<br>- The image of the farmer is redefined as a regional supplier close to nature and oriented towards services. A new label for fair regional products supports this change.<br>- The Alpine ascent and descent of the cattle event (‘Alpauf- und Alpabzüge’) is valued commercially successfully.
Settlement	Villages and hamlets are active all year around.<br>Villages are preserved and managed with reduced secondary residences.<br>Accesses to land and ownership are facilitated.<br>The number of residential services has increased, including access to health care.<br>Access to digitised work environment for teleworking and co-working is guaranteed.<br>The waste is valorised locally and pollution is limited.	Villages have been preserved and modernized.<br>The expansion of Lonza has contributed significantly, but also a more flexible digitized work environment allowing more people from other regions to live and work temporarily in the valleys. These self-employed workers find perfect working conditions in both valleys, such as temporary offices and short-term accommodation, as well as broadband internetaccess and effective digitization.
Demography	Population growth within the limits of the region's capacity, and change in community structure (more permanent residents and workers).<br>The region attracts a new type of workers linked to teleworking.	Population continued to grow.<br>Change in community structure due to a much larger number of residents and temporary workers.
Forestry	No forestry (no perceived opportunity related to ongoing forest regrowth).	The wood of the region, as a precious raw material is increasingly used, for example in the construction of houses and furniture by local craftsmen. This

		promotes local identity, jobs and green building, as well as the preservation of local forests and thus the preservation of an attractive landscape.
Mobility	Outside accessibility and inside mobility are improved.<br>Soft, shared, car free and green mobility (on foot, by bike, electric bus, carpooling etc.) are favoured and supported by municipalities and regions.	Integrated electric mobility:<br>- Secure and environmentally responsible accessibility is in place to and from the valleys.<br>- Mobility is electric.<br>- Safety is greatly enhanced by autonomous vehicles.<br>- The valleys are perfectly connected in 20 minutes by public transport.<br>- There is more cycling infrastructure, especially e-bikes make an integral part of the regional mobility offer.<br>Controlled protection against natural hazards – 24/7 secured access:<br>- The valleys and especially their access are 100% protected against natural hazards such as landslides and avalanches. This security was ensured by the construction and tunnelling of traffic routes, as well as by a new cable car.<br>The blue areas, recently reviewed, are systematically respected during construction work. Few relocations are necessary.
Energy	Energy self-sufficiency through glacier-reliant hydropower and solar energy is achieved.	The glaciers of Saas and Mattertal are the guarantors of hydropower. Wind energy is therefore visually absent.<br>Solar cells, installed on all south-facing roofs, make an integral part of the autonomous supply of renewable energy.
Economy	Tourism is the spine of the local economy supporting the development of other sectors.<br>New job opportunities brought by teleworking, as well as new product distribution networks and markets.<br>Economic performance is of low importance.	Local crafts and regional products are clearly recognizable in 2040 by an established and commercially recognized label. They contribute greatly to regional economic activity.
Governance	Cooperation, participation and consultation for local decisions and economic development.<br>The region benefits from a rich social fabric, sharing, solidarity among people. Haut-Romanche is a “terre d’accueil”.	Cooperation and networking:<br>- The Saas Valley and the Matter Valley are indissociable and economically connected. The two valleys jointly manage their development. Visp is the central hub. All valley communities have found a way to make key decisions together.

Illustrations of visioning process and graphical outputs

Visioning process for one group in Haut-Romanche

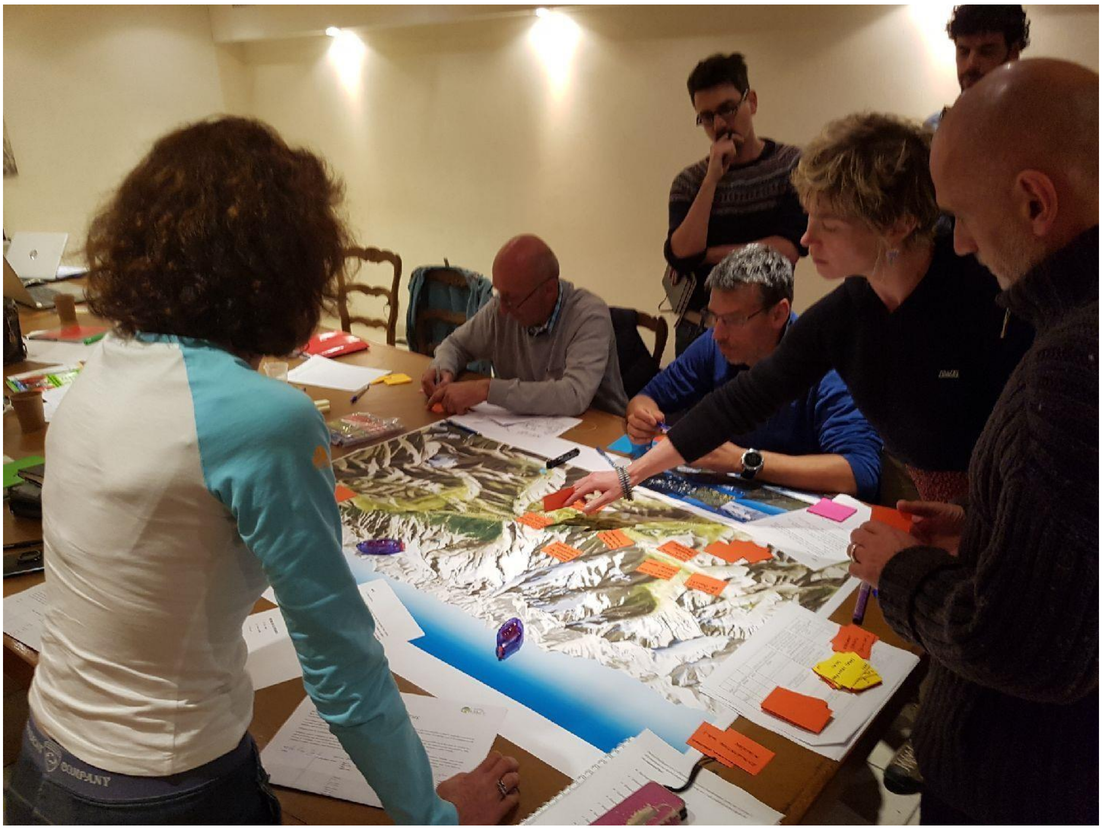

Graphical outputs for one group from a visioning workshop in Haut-Romanche

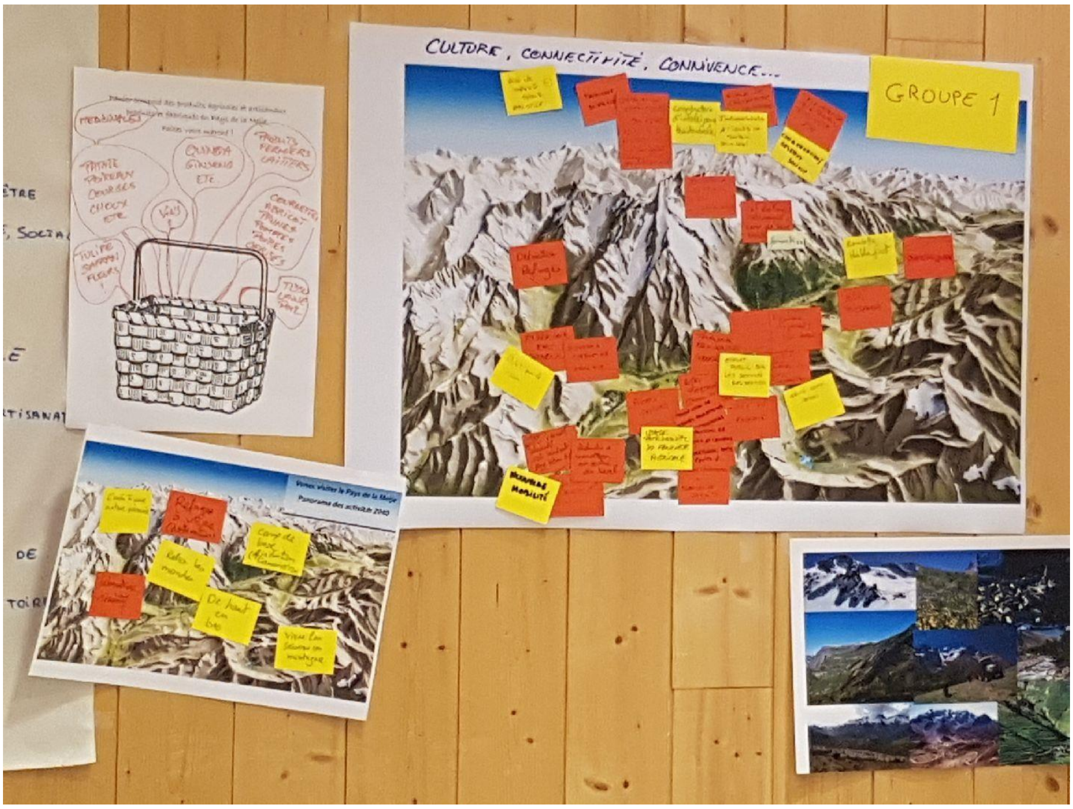

Graphical outputs created during one workshop in the Visp district, for three groups

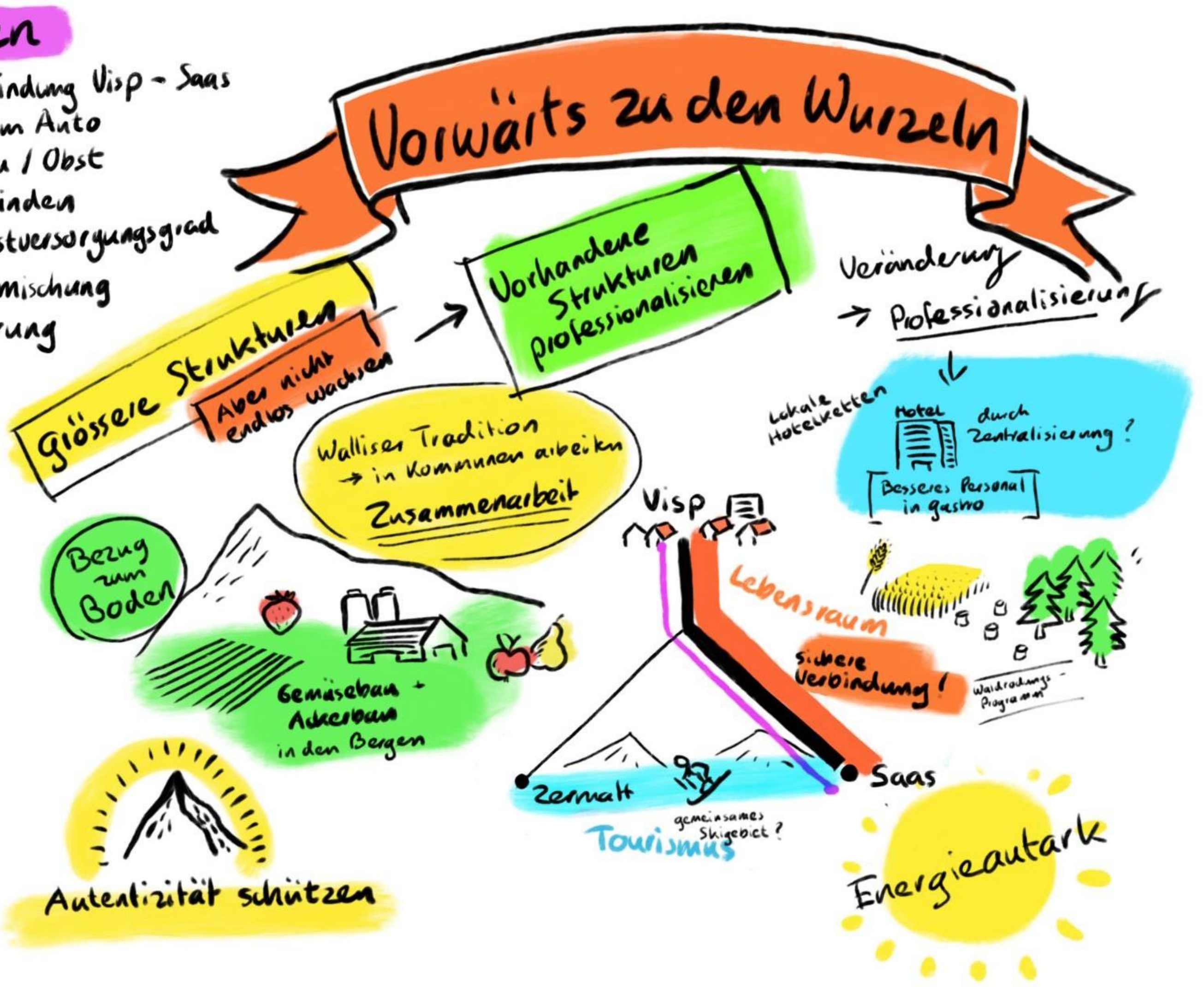

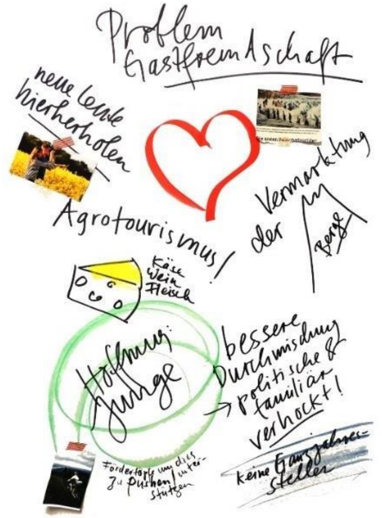
Problem Gastfreundschaft
neue Leute hierherholen
Agrotourismus!
Vermarktung der
Käse Wein Fleisch
Hoffnung: Junge
bessere Durchmischung politische & familiär verhockt!
keine Ganzjahresstellen

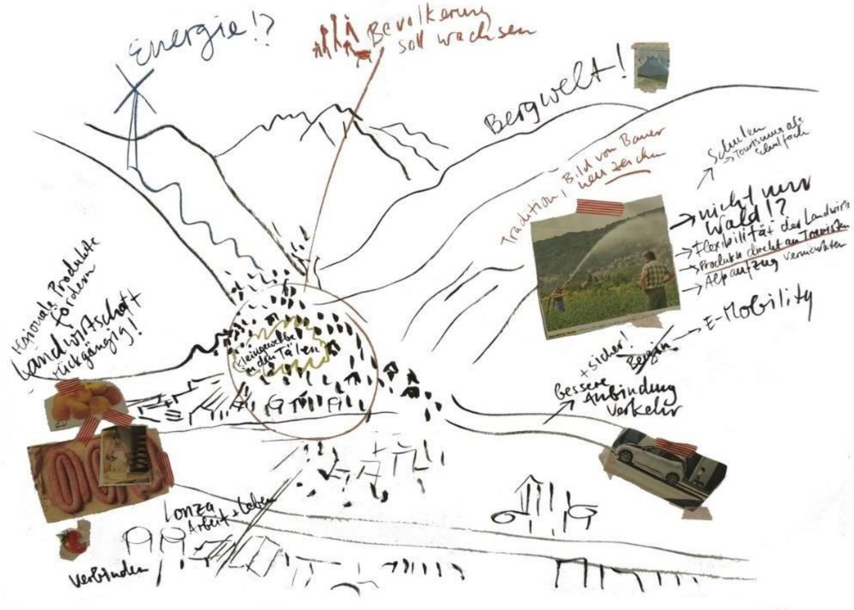
Energie!?
Bevölkerung soll wachsen
Bergwelt!
Tradition, Bild vom Bauer neu zeichnen
Schulen
nicht nur Wald!?
Flexibilität der Landwirtschaft
Produkte direkt an Touristen
Alpaufzug
E-Mobility
Regionale Produkte fördern
Landwirtschaft rückgängig!
Bessere Anbindung Verkehr
Lonza Arbeit + Leben
Verbunden

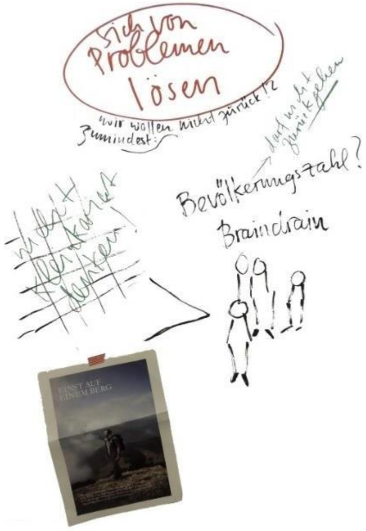
Sich von Problemen lösen
Wir wollen nicht zurück
Bevölkerungszahl?
Braindrain

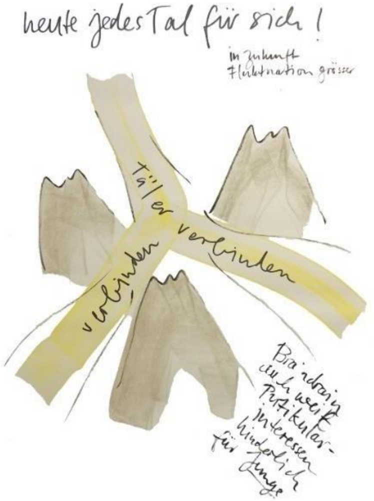
heute jedes Tal für sich!
Täler verbinden
verbinden
Braindrain

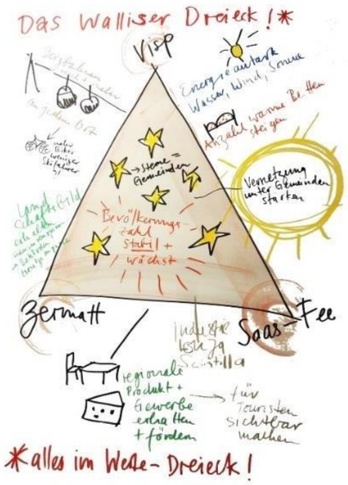
Das Walliser Dreieck!*
Visp
Energieautark Wasser, Wind, Sonne
Anzahl warme Betten steigen
Vernetzung unter Gemeinden stärken
Bevölkerungszahl stabil + wächst
Zermatt
Saas-Fee
regionale Produkt + Gewerbe erhalten + fördern
für Touristen sichtbar machen
*alles im Walli-Dreieck!

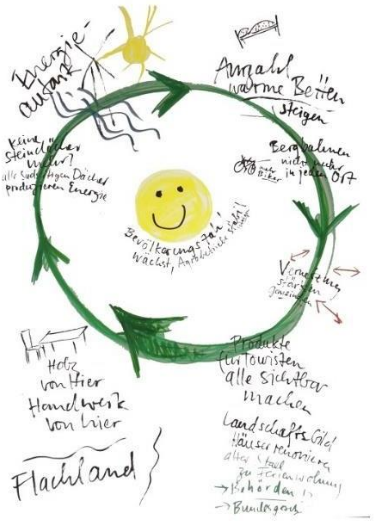
Energieautark
Anzahl warme Betten steigen
Bergbahnen
Bevölkerungszahl wächst
Holz von Hier
Handwerk von hier
Produkte für Touristen alle sichtbar machen
Landschaftsbild
Flachland

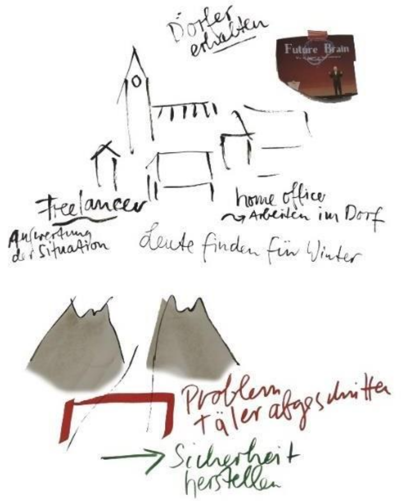
Dörfer erhalten
Future Brain
Freelancer
home office Arbeiten im Dorf
Leute finden für Winter
Problem Täler abgeschnitten
Sicherheit herstellen

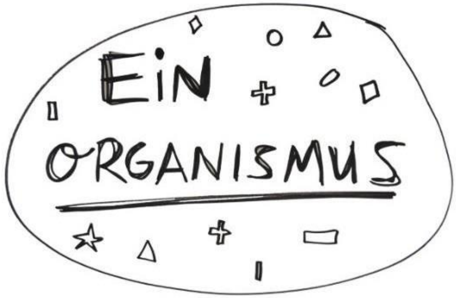

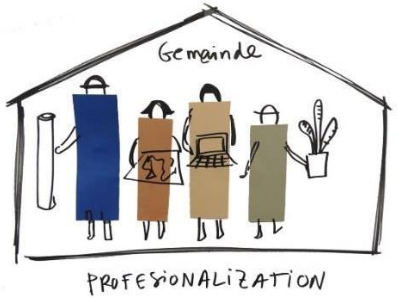

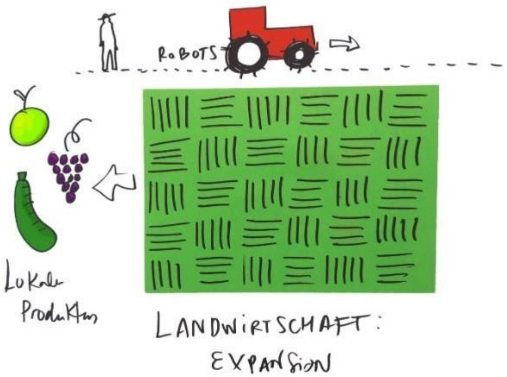

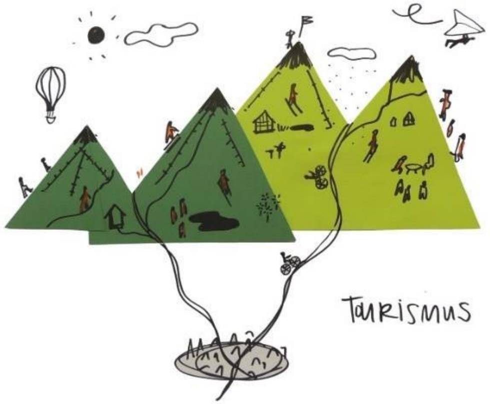

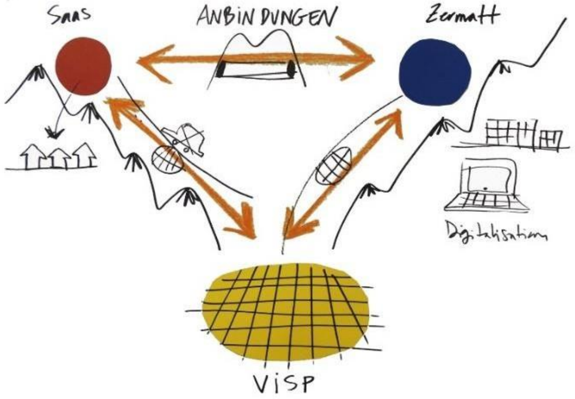

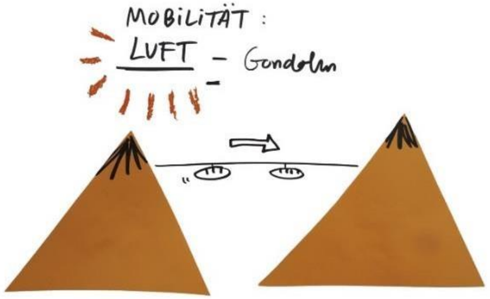

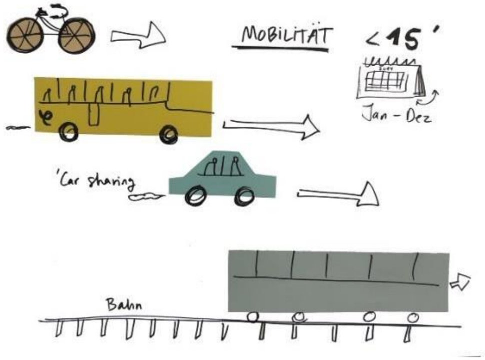

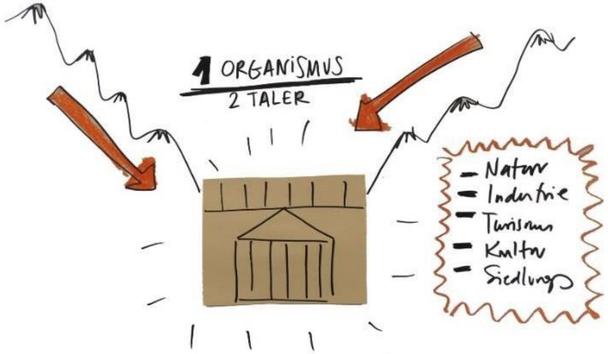

Indikatoren

- Reisezeit < 20'
- 2-Wohnungsanteil ↓ } ~ 60'000 Bett
- Bevölkerungszahlen ↑ }
- Waldfläche ↓ / LW-Fläche ↑ → stabil
- Verhältnis Viehwirtschaft ↓ / Gemüseanbau ↑
- Bevölkerungspyramide
- Selbstversorgungsgrad ↑
- IOT → Wertschöpfung ↑

Table C.2. Vision innovativeness comparison.

	Vision Haute-Romanche	Vision Visp district
Landscape	Uninnovative	Locally innovative
Tourism	Globally innovative	Locally innovative
Agriculture	Uninnovative	Uninnovative
Settlement	Locally innovative	Locally innovative
Demography	Locally innovative	Locally innovative
Forestry	Uninnovative	Locally innovative
Mobility	Uninnovative	Globally innovative
Energy	Uninnovative	Uninnovative
Economy	Globally innovative	Uninnovative
Governance	Uninnovative	Locally innovative

Uninnovative | Locally innovative | Globally innovative
